# Homology Modeling of the Human P-glycoprotein (ABCB1) and Insights into Ligand Binding through Molecular Docking Studies

**DOI:** 10.3390/ijms21114058

**Published:** 2020-06-05

**Authors:** Liadys Mora Lagares, Nikola Minovski, Ana Yisel Caballero Alfonso, Emilio Benfenati, Sara Wellens, Maxime Culot, Fabien Gosselet, Marjana Novič

**Affiliations:** 1 Theory Department, Laboratory for Cheminformatics, National Institute of Chemistry, 1000 Ljubljana, Slovenia; nikola.minovski@ki.si; 2 Jožef Stefan International Postgraduate School, 1000 Ljubljana, Slovenia; ana.caballero@marionegri.it; 3 Laboratory of Environmental Chemistry and Toxicology, Department of Environmental Health Sciences, Istituto di Ricerche Farmacologiche “Mario Negri”—IRCCS, 20156 Milano, Italy; emilio.benfenati@marionegri.it; 4 Laboratoire de la Barrière Hémato-Encéphalique (LBHE), University Artois, UR 2465, F-62300 Lens, France; sara_wellens@ens.univ-artois.fr (S.W.); maxime.culot@univ-artois.fr (M.C.); fabien.gosselet@univ-artois.fr (F.G.)

**Keywords:** P-glycoprotein, molecular docking, protein homology modeling, caco-2 pump out assay

## Abstract

The ABCB1 transporter also known as P-glycoprotein (P-gp) is a transmembrane protein belonging to the ATP binding cassette super-family of transporters; it is a xenobiotic efflux pump that limits intracellular drug accumulation by pumping the compounds out of cells. P-gp contributes to a decrease of toxicity and possesses broad substrate specificity. It is involved in the failure of numerous anticancer and antiviral chemotherapies due to the multidrug resistance (MDR) phenomenon, where it removes the chemotherapeutics out of the targeted cells. Understanding the details of the ligand–P-gp interaction is therefore crucial for the development of drugs that might overcome the MRD phenomenon and for obtaining a more effective prediction of the toxicity of certain compounds. In this work, an in silico modeling was performed using homology modeling and molecular docking methods with the aim of better understanding the ligand–P-gp interactions. Based on different mouse P-gp structural templates from the PDB repository, a 3D model of the human P-gp (*h*P-gp) was constructed by means of protein homology modeling. The homology model was then used to perform molecular docking calculations on a set of thirteen compounds, including some well-known compounds that interact with P-gp as substrates, inhibitors, or both. The sum of ranking differences (SRD) was employed for the comparison of the different scoring functions used in the docking calculations. A consensus-ranking scheme was employed for the selection of the top-ranked pose for each docked ligand. The docking results showed that a high number of π interactions, mainly π–sigma, π–alkyl, and π–π type of interactions, together with the simultaneous presence of hydrogen bond interactions contribute to the stability of the ligand–protein complex in the binding site. It was also observed that some interacting residues in *h*P-gp are the same when compared to those observed in a co-crystallized ligand (PBDE-100) with mouse P-gp (PDB ID: 4XWK). Our in silico approach is consistent with available experimental results regarding P-gp efflux transport assay; therefore it could be useful in the prediction of the role of new compounds in systemic toxicity.

## 1. Introduction

The ATP-binding cassette (ABC) transporter ABCB1, known as P-glycoprotein (P-gp) is a transmembrane efflux transporter with a broad substrate specificity that limits intracellular drug accumulation and contributes to a decrease of toxicity. It is present in normal tissues linked to excretory or barrier functions, as well as in tumor cells, where it is responsible for resistance to a large variety of chemotherapeutic drugs; a phenomenon known as the multidrug resistance (MDR) [1]. The structure of this transporter consists of two symmetrical and homologous halves that act in a coordinated manner as a unit, each with six transmembrane domains (TMD) and a nucleotide binding domain (NBD) located on the cytosolic surface [2] responsible for ATP binding and hydrolysis. 

P-gp can interact with large numbers of structurally diverse compounds, which according to their interactions can be classified as substrates, inhibitors and modulators [3]. Compounds actively transported by P-gp are known as substrates, whereas those that compromise the transporting function of P-gp are known as inhibitors. Modulators interact with P-gp reducing substrate binding through a negative allosteric modulation. 

Studies have been done trying to understand the nature of the P-gp ability for binding so many different compounds and to elucidate the attributes of the drug binding pocket. A study made via photoaffinity labelling of P-gp with azidopine showed that there are two different binding sites for this drug [4]. In the successive years, three distinct binding sites have been suggested: the H-site interacting with Hoechst 33342 and colchicine, R-site interacting with rhodamine 123 (R123) and anthracyclines, and a third binding site exerting allosteric interaction with the previous two [5]; over time the number of binding sites has increased up to seven [5]. In addition, the “substrate induced-fit” mechanism has also been proposed suggesting that a substrate, depending on its size and shape, is able to induce conformational changes in the transmembrane (TM) segments, allowing the substrate to accommodate within P-gp and successively be transported [6]. 

Due to the importance of P-gp on MDR and absorption, distribution, metabolism, excretion, and toxicity (ADMET) properties, many studies have been conducted with the aim of identifying P-gp substrates and developing more effective P-gp inhibitors [7]. For this purpose in silico models have been recognized to be valuable tools [8,9] and the methodologies employed are either ligand-based or structure-based prediction methods [10]. Quantitative structure–activity relationship (QSAR), a traditional ligand-based method, has been extensively applied in predicting the biological activity providing rapid and cost-effective screening platforms for identifying P-gp inhibitors or substrates. A P-gp classification model was also developed in the authors’ previous study [11]. On the other hand, structure-based methods (e.g., molecular docking) allow the investigation of ligand–receptor interactions at atomistic level when high-resolution structures of the receptors are available. Until 2019, when the cryoEM structure of human ABCB1 was resolved (PDB ID: 6QEX) [12], the docking studies on P-gp for understanding the binding site interaction profiles had been limited due to the availability of the experimentally solved structure of human P-gp (*h*P-gp) [13,14,15], thus the use of homology models became necessary for studying the ligand–*h*P-gp interactions. 

The first homology models developed and utilized in molecular docking studies relied on bacterial homologues used as templates, such us the bacterial transporters Sav1866 and MsbA structures [16,17,18], representing different catalytic states of the transport cycle. In 2009, the crystal structure of the mouse P-gp (*m*P-gp) complex with a cyclic tetrapeptide (PDB ID: 3G5U) [19] was resolved, hence representing a ligand binding competent conformation of the protein. The *m*P-gp, with 87% sequence identity is a well-suited template for homology modeling of the *h*P-gp and provides a better model for structure-based approaches.

In the present study, we developed a *h*P-gp homology model based on *m*P-gp multiple templates that can be used in further docking simulations. We used the available knowledge on the interaction of substrates and inhibitors with P-gp to apply a molecular docking approach in order to first elucidate if molecular docking is able to differentiate between active and non-active P-gp compounds; and second, for determining to what extent the amino acids predicted by molecular docking are consistent with experimental data available. For this purpose, we have carried out molecular docking simulations on a set of thirteen compounds, which belong to the classes mentioned above, and the homology model of *h*P-gp. Ligand–protein binding energies, number and type of interactions were analysed in order to assess if there are significant differences between the compounds under study.

## 2. Results and Discussion

### 2.1. Homology Modeling of hP-gp

The crystal structure of *m*P-gp (PDB ID: 4M1M), which has an 87% sequence identity to the *h*P-gp, was selected as the most suitable template for developing the homology models of the 3D structure of *h*P-gp. However, the models built with Discovery Studio Client/Modeler 9.12 [20,21] and I-TASSER [22,23,24] tools were based on the alignment utilizing more than one template, among these the crystal structure of *Caenorhabditis elegans* P-gp (PDB ID: 4F4C), included in the model generated with the Discovery Studio 4.1 Client/Modeler tool 9.12 [20,21]. 

The alignments between the *h*P-gp sequence and the templates used are provided in the Supplementary materials, Figure S1. Several models were built with each of the tools and one model per tool was selected for further evaluation and validation. 

#### 2.1.1. SWISS-MODEL

**Figure 1:**
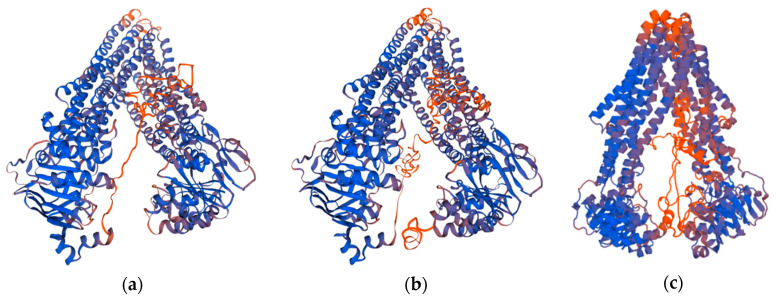
Three-dimensional (3D) structures of the *h*P-gp models selected. The models are presented in colors based on the QMEAN model quality values to allow instant visualisation of regions of the model that are well (blue) or poorly (orange) modeled: (**a**) Model generated by SWISS-MODEL tool; (**b**) model generated by I-TASSER tool; (**c**) model generated by Discovery Studio 4.1 Client/Modeler 9.12 tool.

**Figure 2:**
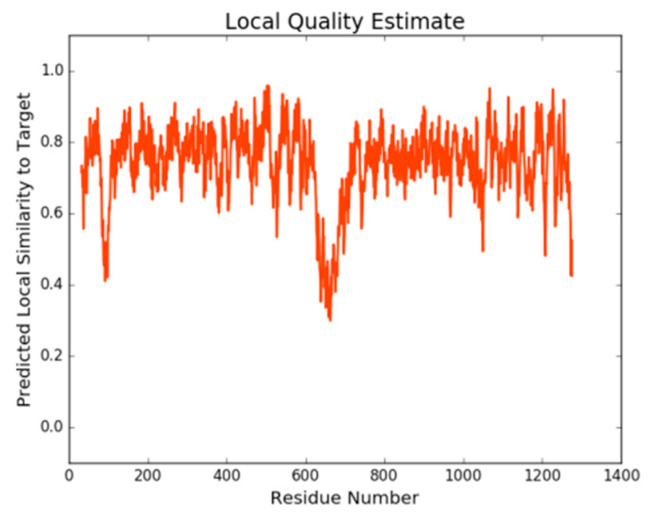
Local quality plot of the Model 1, generated by the SWISS-MODEL tool.

A total of five initial *h*P-gp models were created with the SWISS-MODEL tool [25] using as template the crystal structure of the *m*P-gp (PDB ID: 4M1M). The obtained models were scored using the GMQE (global model quality estimation) and QMEAN [26] scoring functions. The Model 1 (Figure 1a) was selected for further evaluation and validation as it shows the best quality factors in the set of models developed (Table 1). Even though, in general terms, the selected 3D structure was assessed as a good quality model, some regions are yet poorly modeled and the residues involved can be easily identified looking at the Local Quality Plot in Figure 2. According to this plot, a majority of the residues in the structure are in good agreement with the estimated native structure, apart from the residues belonging to the linker region (a disorganized coil region of approximately 75 residues long). These residues were modeled with less reliability as their individual QMEAN scores are below the threshold value of 0.6. One of the reasons why this region is less reliably modeled could be because the linker region between the two homologous halves of the protein has not been resolved yet in any of the P-gp crystal structures available; therefore, it is not present in any of the templates used. 

#### 2.1.2. I-TASSER

**Figure 3:**
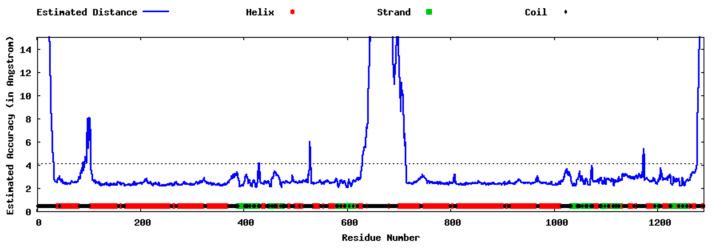
Local structure error profile of the Model 1 generated by the I-TASSER tool.

Five models were generated by the I-TASSER tool using as template the crystal structures of *m*P-gp (PDB IDs: 4M1M, 5KO2, 5KOY, 3G61, 3G5U). The resulting models were scored using C-score, TM-score [27], and root mean square deviation (RMSD) scoring functions. The C-scores for the five models generated are reported in Table 2, while the TM-score and RMSD are reported just for the Model 1. The correlation between C-score and TM-score is weak for lower rank models; thus, they are not calculated. However, the C-score, Number of decoys and Cluster density for all models are reported for a reference. The Model 1, shown in Figure 1b, was selected for further evaluation and validation as it presents the best quality factors in the set of models generated; a positive C-Score value of 0.49, a high TM-Score value of 0.78 and the largest cluster size. From the Estimated Local Accuracy Plot shown in Figure 3, it can be noticed that the residues belonging to the linker region have a bigger distance deviation (in Angstrom) between the residue positions in the model and the predicted native structure. In Figure 1b, the poorly predicted regions of the Model 1 can also be observed; it can be noticed that these regions correspond with those assessed in the previous model (Figure 1a). As mentioned before, a reason why the linker region is less reliably modeled could be the lack of crystallographic information regarding it in the P-gp crystal structures available. 

#### 2.1.3. Discovery Studio/Modeler

**Figure 4:**
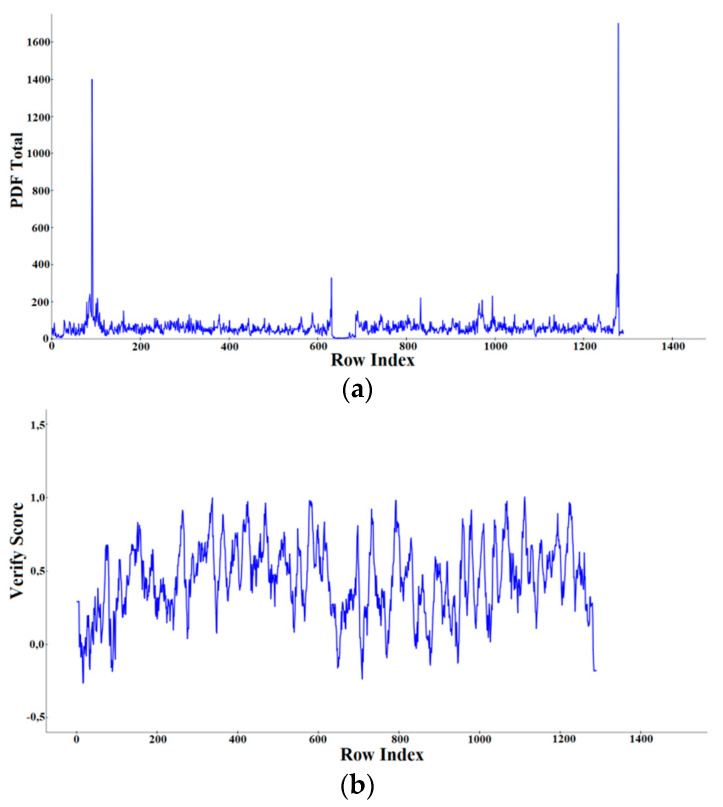
(**a**) PDF Total Energy Plot; (**b**) Verify Score Plot of the Model 16 generated by Discovery Studio 4.1 Client/Modeler 9.12 tool.

A total of 20 initial models were generated by Discovery Studio 4.1 Client/Modeler 9.12 using as template the crystal structures of *m*P-gp (PDB IDs: 6FN4, 4M1M, 5KPI, 4M2S, 5KO2, 3G60) and the crystal structure of *C. elegans* P-gp (PDB ID: 4F4C). The resulting models were scored using the the Discrete Optimized Protein Energy (DOPE) score and PDF Total Energy scoring functions (Table 3). As all the models have similar Probability Density Function (PDF) Total Energy, the DOPE score was then used for selecting the best-ranked model. The Model 16 (Figure 1c) was selected for further evaluation via the Verify Protein protocol in Discovery Studio 4.1 Client. According to the scores presented in Table 4, the model 16 is of good quality, as the verify score obtained is higher than the Verify Expected Low Score value and very close to the Verify Expected High Score value. The areas of the structure with large violations of the homology restraints are shown in Figure 4a by means of the PDF Total Energy plot. The verify score per amino acid which indicates whether a residue is in the desired 3D environment or not is shown in Figure 4b. The regions of the protein over which the score approaches zero or becomes negative are likely to be misfolded and should be carefully examined. Additionally, in this case, the less reliable areas of the model correspond with those found in the previous models (Figure 1a,b). 

### 2.2. Models Validation

The quality of the models was assessed in order to verify that they are reliable and suitable for carrying out further molecular docking simulations. The validation of the selected models from each modeling tool was done utilizing available online quality structure assessment tools, such as PROCHECK [28], Verify 3D [29], ERRAT [30], and PROVE [31]. 

**Figure 5:**
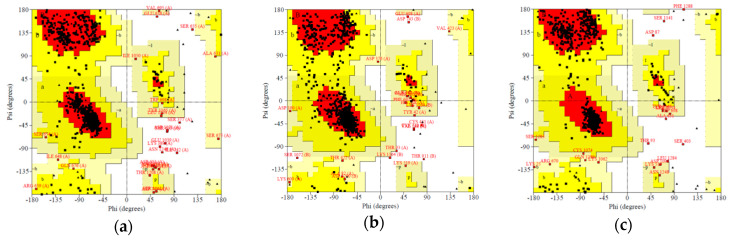
Ramachandran Plots for the modeled 3D structures of the *h*P-gp. The red, yellow and white areas represent the favored, allowed and disallowed regions respectively: (**a**) model generated by SWISS-MODEL tool; (**b**) model generated by I-TASSER tool; (**c**) model generated by Discovery Studio 4.1 Client/Modeler 9.12 tool.

The stereochemical properties of the *h*P-gp models were evaluated using the PROCHECK software via the Ramachandran Plot and the results were compared with those obtained from the crystal structure of *m*P-gp (PDB ID: 4M1M). The resulting Ramachandran Plots of the predicted *h*P-gp models are shown in Figure 5 and the related statistics are reported in Table 5. 

The plots revealed that the phi (φ) and psi (ψ) backbone dihedral angles in the *h*P-gp models are reasonably accurate as a majority of the residues are inside the allowed regions; less than 1.0% of the residues are in the disallowed regions in all the models evaluated. The residues in the disallowed region are located mainly in the NBDs of the protein, apart from one residue (Y710) in the I-TASSER model, which is located in the TM domain helix; however, none of the residues of the binding pocket are in the disallowed regions. Considering the φ/ψ distribution of the amino acids in the modeled *h*P-gp structures, the results are consistent with those obtained from the experimentally available *m*P-gp structure (PDB ID: 4M1M) reported in Table 5. In summary, the stereochemical quality of the models is satisfactory, with similarities to that of the template used. 

**Figure 6:**
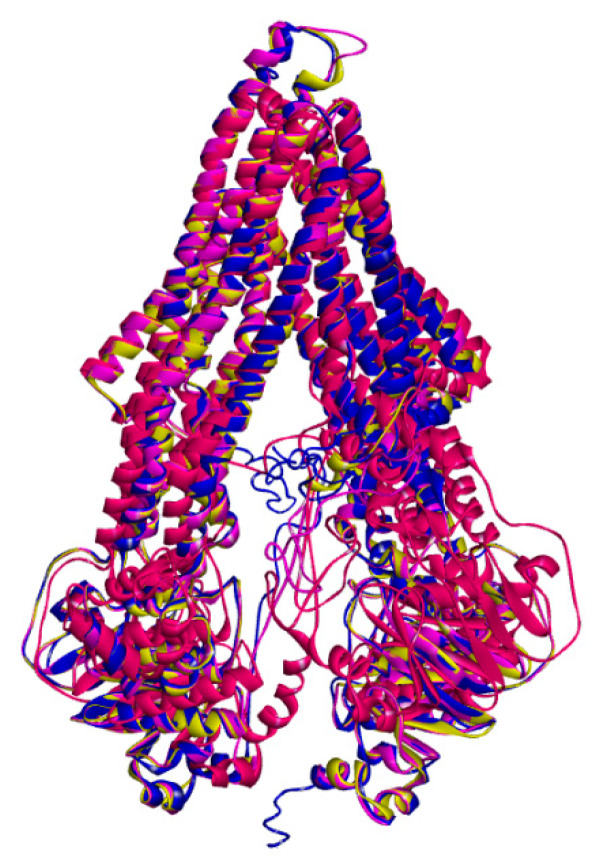
Superimposed protein structures of the *h*P-gp models generated and the crystal structure of *m*P-gp (PDB ID: 4M1M). The *m*P-gp is colored in yellow.

In order to assess the overall folding of the models, a structural comparison of the *h*P-gp models developed was done in Discovery Studio 4.1 by superimposing the *h*P-gp models over the crystal structure of the *m*P-gp (PDB ID: 4M1M) (Figure 6). The main-chain root mean square deviation (RMSD) and the Number of Overlapping Residues are shown in Table 6. The RMSD values with respect to the side chain, alpha carbons, and the whole protein were also calculated and are shown in Table 7.

The results demonstrated a small Main-chain RMSD against the crystal structure of *m*P-gp (PDB ID: 4M1M) with values of 0.24 Å and 0.70 Å for SWISS-MODEL and I-TASSER models, respectively. The model generated with Discovery Studio 4.1 Client/Modeler 9.12 resulted in the largest RMSD value of 1.83 Å. When a model is overlapped to the template, the generally accepted RMSD threshold is of 2.0 Å; thus, the three *h*P-gp models assessed are within the accepted limit. The superimposition between the *m*P-gp (PDB ID: 4M1M) and *h*P-gp models revealed that the models developed exhibit significant 3D similarities and that the overall folding is correct. 

Additional analysis of the predicted *h*P-gp 3D structures was done using Verify 3D, ERRAT and PROVE structural assessment tools (Table 8). The Verify 3D tool, which provides an analysis of the compatibility of the 3D models with their amino acid sequence (1D), resulted in scores smaller than 80% for the three models assessed. This means that less than the 80% of the amino acids in the structures have a score ≥0.2 in the 3D/1D profile. The best score was obtained for the I-TASSER model with 63.41% of the residues with averaged 3D/1D score ≥0.2; very close to the result obtained by the crystal structure of the *m*P-gp (PDB ID: 4M1M). SWISS-MODEL and Discovery Studio models resulted both with 45% of the residues with averaged 3D/1D score ≥0.2. According to these results none of the predicted 3D models of *h*P-gp passed the assessment; however, the quality indicator performed poorly also on the crystallized structure of *m*P-gp with just 65.20% of the residues within the scoring limit. 

The overall quality factor for non-bonded atomic interactions between different atom types in the modeled structures was assessed using the ERRAT program. The ERRAT score is expressed as the percentage of the protein for which the calculated error value falls below the 95% rejection limit. The ERRAT score should be greater than 50% for considering a model of good quality. The overall quality factors were around 95% for SWISS-MODEL and I-TASSER models, being the I-TASSER model the one with the highest score of 96.08%. The Discovery Studio model and the crystal structure of the *m*P-gp (PDB ID: 4M1M) resulted both with scores around 80% below the rejection limit. Based on the previous results, the current 3D *h*P-gp models assessed have good reliability. 

The volume-based structure validation of the *h*P-gp models was done utilizing the PROVE program. The crystal structure of the *m*P-gp (PDB ID: 4M1M), used as a reference for the validation of the models developed, resulted in less than 1% of buried outlier atoms, which is the threshold value for considering the test passed. Outliers are considered here as being those buried atoms for which the volume is more than 3.0 standard deviations away from the expected volume. The results for the three models assessed indicated that there are some errors present in the structures as the percentage of buried outlier atoms was greater than 5% in all the cases. 

The structure quality assessment made using the online tools PROCHECK, Verify 3D, ERRAT, and PROVE suggested that the three *h*P-gp models developed are as good as the crystal structure of the *m*P-gp (PDB ID: 4M1M) used as a template. They are reliable and of suitable quality for further molecular docking simulations. Nonetheless, the I-TASSER *h*P-gp model had a better performance with respect to the other two models in a majority of the tests. 

In order to check any possibility of a bias in the quality of the selected *h*P-gp model (I-TASSER) that comprises NBDs (according to the full primary sequence of the *h*P-gp protein used), an additional quality assessment of its truncated counterpart (without NBDs) was performed (see Table S4, Figure S6). As demonstrated in Table S4, there are no significant differences between both models in terms of calculated quality scores, with a slight exception for Verify 3D score (38.19% for truncated *h*P-gp model comparing to 63.41% for the selected full-length *h*P-gp model). The latter was somehow expected, since Verify 3D scoring method is grounded on the 1D (primary sequence) of the model as well as its secondary-structure composition [29], which structural information are actually missing in the truncated *h*P-gp model. Nevertheless, ERRAT and PROVE scores as well as PROCHECK assessments (Figure S6) suggest that truncated *h*P-gp model (without NBDs) is highly comparable to the selected one in terms of quality and reliability, allowing the use of the selected full-length I-TASSER *h*P-gp model for further structure-based (molecular docking calculations).

### 2.3. Molecular Docking Calculations

#### 2.3.1. Docking into Homology Model

Ligand docking is a commonly used approach to identify ligand–protein interactions. However, in the case of P-gp, this could be challenging due to the high degree of flexibility and the large binding cavity consisting of multiple binding sites [19,32]. In addition to this, P-gp can bind more than one ligand simultaneously [33,34] and until 2019, when the cryoEM structure of human ABCB1 was resolved (PDB ID: 6QEX) [12], there was a lack of a high resolution crystal structure of *h*P-gp which rendered necessary the use of protein homology models, adding additional layers of uncertainty to the process. Nonetheless, a recent study has reported the use of homology models in virtual screening applications with a superior performance in comparison to crystal structures [35]. This fact is explained by the conformational flexibility provided by homology models which allows a better accommodation of diverse ligands and therefore a better screening performance. 

Two docking runs were performed utilizing two different algorithms, CDOCKER [36] and GOLD [37,38]. In order to analyse the binding pocket of the *h*P-gp, the simulations started with the docking of thirteen compounds, among them eight well-known molecules which interact with P-gp as substrates, inhibitors or both: cyclosporin A (CsA), amiodarone (AM), doxorubicin (DOX), digoxine (DIG), loperamide (LPM), rifampin (RMP), verapamil (VER), carvedilol (CAR), and five non-interacting compounds with P-gp: valproic acid (VPA), busulfan (BU), gentamicin (GEN), pamidronate (APD), and paraquat (PQ). The large binding pocket, observed in the *m*P-gp crystal structure (PDB ID: 4M1M), binds the ligands at different sites with partially overlapping residues; some of them identical to those involved in rhodamine and verapamil binding [39,40,41,42]. Therefore, when defining the binding site for performing the docking simulations, the entire transmembrane (TM) region was considered. The binding region was delineated by those atoms within a radius of 15 Å and 24.7 Å, for CDOCKER and GOLD calculations respectively, utilizing the experimental coordinates of the co-crystallized *m*P-gp ligand (PDB ID: 4XWK). The selection of the binding site and the settings for the docking simulations were validated via re-docking (ligand reproduction) procedure, obtaining heavy-atoms RMDS values of 1.5697 Å, 1.6021 Å, 1.6427 Å for CDOCKER calculations and 0.5527 Å, 0.7988 Å, 0.8498 Å for GOLD calculations (Table S2, and Figure S4 in Supplementary Materials). The RMSD results are in agreement with the accepted threshold of 2 Å.

The resulting docking poses were subsequently rescored with fourteen additional scoring functions implemented in Discovery Studio 4.1 Client. The main fitness function used during the docking runs and the rescoring functions calculated for the resulting poses are listed in Table 9.

Based on the sum of ranking differences (SRD) results, the best ranking poses were selected using the consensus ranking scheme, fusing the six best performing scoring fitness functions and using the geometric mean for computing the fused rank (Table 10).

**Figure 7:**
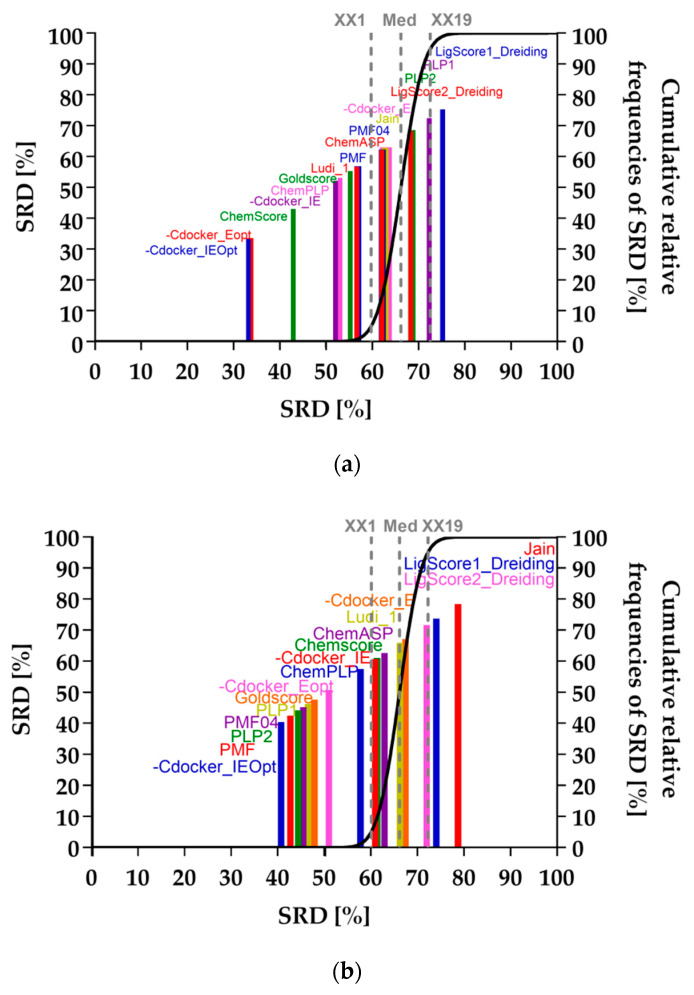
The sum of ranking differences (SRD) analysis of the 16 fitness functions calculated for each docking run: (**a**) CDOCKER; (**b**) GOLD. Normalized SRD values are plotted on the x and left y axes. The cumulative relative frequencies of SRD values for random ranking are plotted on the right y axis and shown as the black curve.

According to the SRD results, the best performing scoring functions for the CDOCKER run presented a very low probability that their performance was of random character, with values greater than 2.91E-15% and smaller or equal to 0.92% (see Table 11); they performed better than random ranking, as they do not overlap with the cumulative relative frequency curve of a random ranking shown in Figure 7a. For the GOLD results, the best performing scoring functions presented a probability of being of random character greater than 2.64%–10% and smaller or equal to 1.01%, as you can observe in Table 12. Additionally, in this case, they do not overlap with the cumulative relative frequency curve of random ranking shown in Figure 7b. 

**Figure 8:**
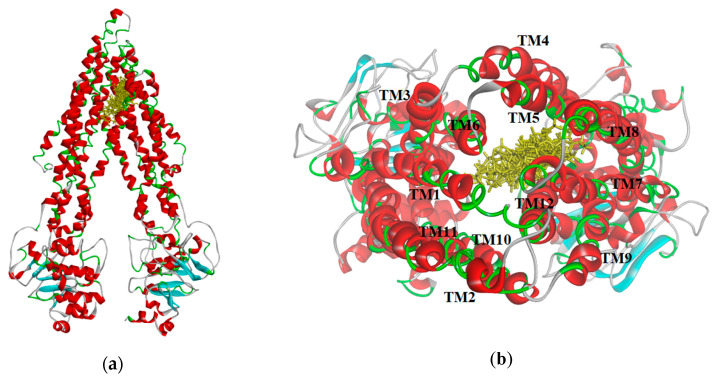
(**a**) Distribution of the selected ligand poses (yellow) in the homology model of *h*P-gp; (**b**) View from the extracellular side of the protein looking into the internal chamber. The colors representation is according to the secondary structure: helices are red, beta sheets are cyan, turns are green, and coils are white.

**Figure 9:**
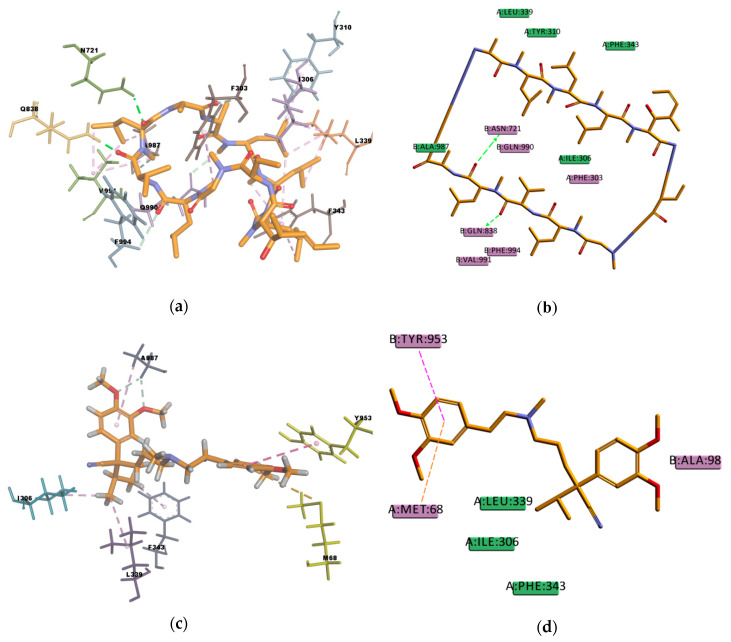
Cyclosporine A (CsA) and verapamil (VER) top-ranked poses obtained with GOLD algorithm. (**a**) 3D view of CsA interactions in the binding pocket. Green dotted lines represent conventional hydrogen bond interactions, light-green dotted lines represent carbon hydrogen bond interactions and light-rose dotted lines represent hydrophobic interactions. Residue Q838 (TM 9) is highlighted in yellow; (**b**) 2D interaction diagram of CsA with *h*P-gp interacting residues. The green dotted lines represent conventional hydrogen bond interactions; (**c**) 3D view of VER interactions in the binding pocket. Residues M68 (TM1) and Y953 (TM11) are highlighted in yellow. Light-green dotted lines represent carbon hydrogen bond interactions, light-rose dotted lines represent hydrophobic interactions, the pink dotted line represents a π–π stacking interaction, and the orange dotted line represents a π–sulphur interaction; (**d**) 2D interaction diagram of VER with *h*P-gp interacting residues. The pink dotted line represents a π–π stacking interaction with the residue Y953, and the orange dotted line represents a π–sulphur interaction with the residue M68.

The resulting poses were distributed within the TM regions of P-gp (Figure 8), showing interactions with protein residues of multiple TM helices located throughout the binding region. Interacting amino acids were identified with the tool “ligand interactions” in Discovery Studio 4.1 Client. For the CDOCKER results, residues primarily located on TM helices 5, 6, 7, 8, 10, and 12 were involved in binding, while for the GOLD results additionally residues in TM 1, 9, and 11 were involved in the binding of VER and CsA. The pose obtained for CsA is showing one conventional hydrogen bond with Q838 (TM9) and the pose obtained for VER is showing one π–sulphur interaction with M68 (TM1) and one π–π interaction with Y953 (TM11) (Figure 9). 

The docking results obtained with the CDOCKER algorithm were comparable with those obtained with the GOLD algorithm in terms of calculated binding energies, as well as type of interactions between the docked compounds and *h*P-gp. The estimated binding energies for the docking set, calculated using the Calculate Binding Energies protocol in Discovery Studio 4.1 and shown in Table 13, are in close agreement in the two methodologies employed. The type of interactions was essentially the same in both cases (Table 14, Table S1); mainly hydrophobic π–sigma, π–alkyl, and π–π type of interaction with the presence of some hydrogen bond interactions. 

**Figure 10:**
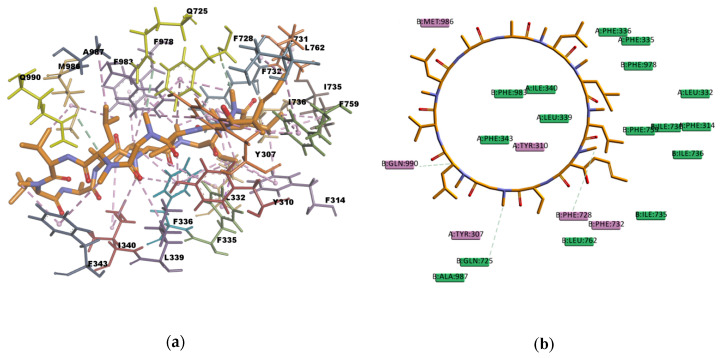
Cyclosporine A (CsA) top-ranked pose obtained with CDOCKER algorithm. (**a**) 3D view of CsA interactions in the binding pocket. Light-green dotted lines represent carbon hydrogen bond interactions and light-rose dotted lines represent hydrophobic interactions. Residues Q990, Q725, and F728 involved in hydrogen bond interactions are highlighted in yellow; (**b**) 2D diagram of CsA with *h*P-gp interacting residues. Light-green dotted lines represent carbon hydrogen bond interactions.

**Figure 11:**
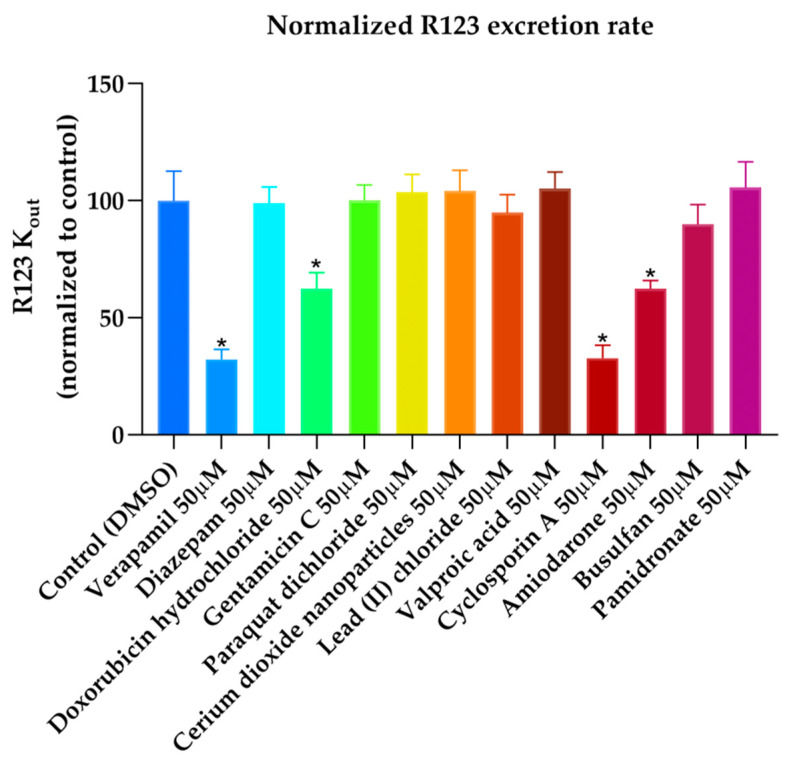
Effects of drugs on the rate of excretion of rhodamine 123 (R123) out of Caco-2 (*n* = 8, mean ± SD, * *p* < 0,0001). Results are expressed as percentage compared to the rate of excretion of R123 in the absence of drug (i.e control DMSO). Error bars: SD; verapamil: positive control; diazepam: negative control.

Based on the visual inspection of the selected docking poses, the interactions between the ligands and residues in the binding pocket are mainly of hydrophobic character. In the case of CsA, there are at least 20 residues involved in hydrophobic interactions (Y307, Y310, F314, L332, F335, F336, L339, I340, I343, F728, I731, F732, I735, I736, F759, L762, F978, F983, M986, and A987) which are reported in Table 13 and can be seen in Figure 10. CsA, though a big molecule, was found to have the lowest binding energy (‒268.516 kcal/mol) in the set of docked molecules. The stability of CsA in the binding site could be attributed to the large number of π interactions present, such as π–alkyl interactions, and to the simultaneous presence of hydrogen bonds in the binding pose. Even if the three hydrogen bonds present in the docked pose are carbon hydrogen bonds type C–H…O, i.e., weak interactions with a greater dispersive component, they may also play a role in stabilizing the ligand–protein complex. CsA is known to be a high affinity substrate of P-gp [43,44], hence the docking results obtained are in agreement with the available literature and with the experimental transport assay results reported in Figure 11, where CsA leads to the lower excretion rate of rhodamine 123 (R123) out of Caco-2 cells. Nevertheless, it should be noted that Caco-2 cells also express other ABC transporters such as ABCG2 and ABCC1 which participate in the active efflux of R123 out of the cells and could be inhibited by CsA as these transporters share many similarities with P-gp.

**Figure 12:**
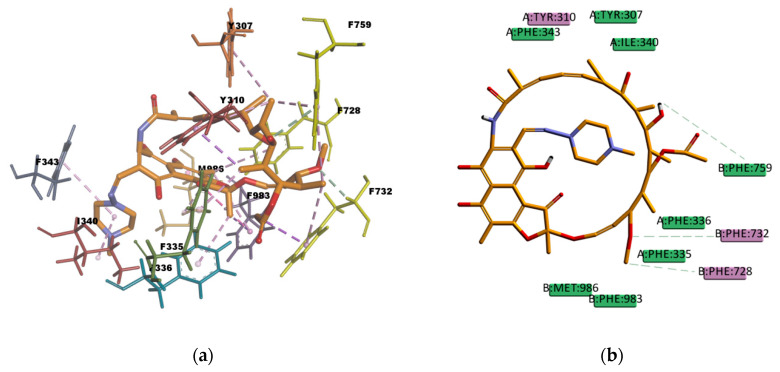
Rifampin (RMP) top-ranked pose obtained with CDOCKER algorithm. (**a**) 3D view of RMP interactions in the binding pocket. Light-green dotted lines represent weak hydrogen bond interactions and light-rose dotted lines represent hydrophobic interactions. Residues F732, F759, and F728 involved in hydrogen bond interactions are highlighted in yellow; (**b**) 2D interaction diagram of RMP with *h*P-gp interacting residues. Light-green dotted lines represent weak hydrogen bond interactions.

Similar as CsA, RMP is a big molecule as well, with a very favorable estimated binding energy of -214.872 kcal/mol. The docking pose reported in Figure 12 shows hydrophobic interactions with 11 residues in the binding pocket (I340, M986, Y310, F732, Y307, F335, F336, F343, F759, F983, and F728. RMP presents a high number of π interactions in its binding mode, among them π–sigma, π–alkyl, and π–π interactions. Hydrogen bond interactions of weak character are also present in the binding pose, such as weak carbon hydrogen bonds, and one π–donor hydrogen bond between the hydroxyl group (donor) in RMP and the π electron cloud over the aromatic ring in F759 (acceptor). The sum of these interactions undoubtedly creates a strong cohesive environment, thereby stabilizing the complex formed. The docking results obtained are in agreement with experimental transport assay results reported in reference [45] and with the literature available regarding RMP and P-gp interactions. RMP is known to be a substrate [46] and inducer [47] of P-gp. 

**Figure 13:**
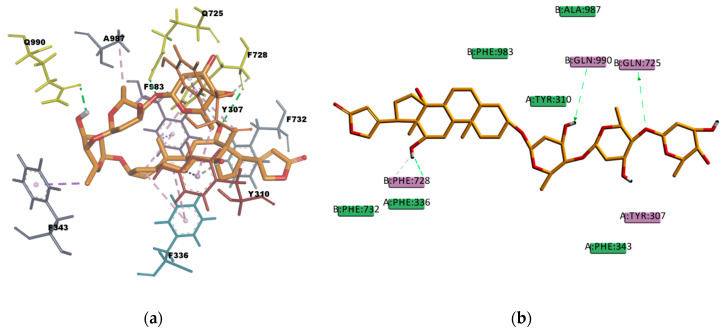
Digoxine (DIG) top-ranked pose obtained with CDOCKER algorithm. (**a**) 3D view of DIG interactions in the binding pocket. Green dotted lines represent conventional hydrogen bond interactions and light-rose dotted lines represent hydrophobic interactions. Residues Q725, Q990, and F728 involved in hydrogen bond interactions are colored yellow; (**b**) 2D interaction diagram of DIG with *h*P-gp interacting residues. The green dotted lines represent conventional hydrogen bond interactions. The light-green dotted line represents a π–donor hydrogen bond interaction.

In Figure 13 the binding pose of DIG can be observed. DIG binding mode involves hydrophobic interactions with eight residues in the binding pocket (A987, F336, F343, F728, F732, F983, Y307, and Y310) and four hydrogen bond interactions. Three of them are conventional, electrostatic N–H…O or O–H…O type, of strong character, and one is π–donor hydrogen bond interaction of weaker character. The sum of these interactions contributes to the stability of the complex which is reflected in the value of the estimated binding energy of -211.755 kcal/mol. This results are in agreement with the literature available that reports DIG as a high affinity P-gp substrate [48]. 

**Figure 14:**
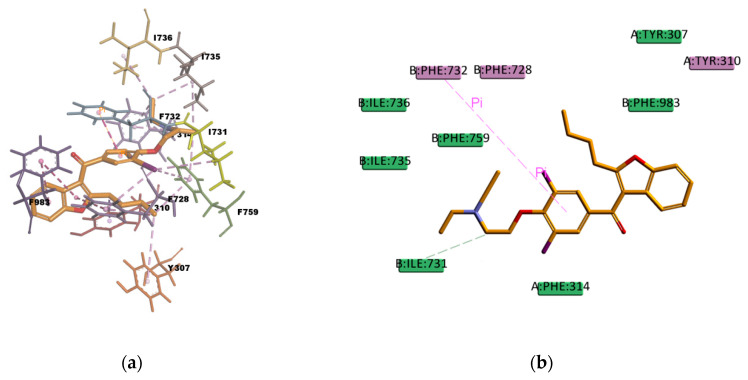
Amiodarone (AM) top-ranked pose obtained with CDOCKER algorithm. (**a**) 3D view of AM interactions in the binding pocket. Light-green dotted lines represent carbon hydrogen bond interactions, light-rose dotted lines represent hydrophobic interactions, and the pink dotted line represents a π–π stacking interaction. Residue I731 involved in a carbon hydrogen bond interaction is highlighted in yellow; (**b**) 2D interaction diagram of AM with *h*P-gp interacting residues. The light-green dotted line represents a conventional hydrogen bond interaction. The pink dotted line represents a π–π stacking interaction with residue F732.

**Figure 15:**
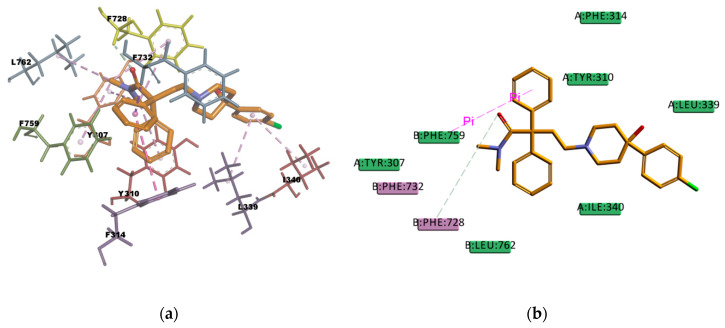
Loperamide (LMP) top-ranked pose obtained with CDOCKER algorithm. (**a**) 3D view of LMP interactions in the binding pocket. Light-green dotted lines represent carbon hydrogen bond interactions, light-rose dotted lines represent hydrophobic interactions, and the pink dotted line represents a π–π interaction. Residue F728 involved in a carbon hydrogen bond interaction is highlighted in yellow; (**b**) 2D interaction diagram of LMP with *h*P-gp interacting residues. The light-green dotted line represents a carbon hydrogen bond interaction, and the pink dotted line represents a π–π interaction with residue the F759.

AM docked pose, shown in Figure 14, presents many hydrophobic interactions involving ten residues in the binding pocket (Y307, Y310, F314, F728, I731, F732, I735, I736, F759, and F983), among them many π–alkyl type of interactions but also π–sigma and π–π type of interactions. The estimated binding energy is favorable but of smaller magnitude compared with CsA or RMP values. The difference in the binding energies could be explained by the lower number of hydrogen bonds in the binding mode; there is only one weak carbon hydrogen bond type of interaction C–H…O with the residue I731. This is also the case of LPM, which shows hydrophobic interactions with nine residues in the binding pocket (L762, F732, Y307, Y310, F728, F759, L339, I340, and F314) but just one weak carbon hydrogen bond type of interaction with the residue F728 (Figure 15). The calculated binding energy of LPM is also favorable but smaller than CsA or RMP energy values, indicating less binding affinity and stability in comparison with them. The results obtained are in agreement with the available literature and with the experimental transport assay results reported in Figure 11 and reference [44] for AM and in reference [45] for LPM. AM and LPM are known substrates of P-gp [44,49,50].

**Figure 16:**
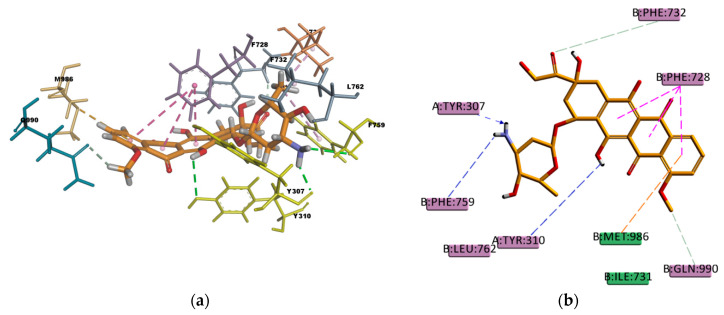
Doxorubicin (DOX) top-ranked pose obtained with CDOCKER algorithm. (**a**) 3D view of DOX interactions in the binding pocket. Green dotted lines represent conventional hydrogen bond interactions, light-green dotted lines represent carbon hydrogen bond interactions, light-rose dotted lines represent hydrophobic interactions, pink dotted lines represent π–π T-shaped interactions, and the orange dotted line represents a π–sulphur interaction. Residues F759, Y307, and Y310 involved in conventional hydrogen bond interaction are highlighted in yellow; (**b**) 2D interaction diagram of DOX with *h*P-gp interacting residues. Green dotted lines represent conventional hydrogen bond interactions, light-green dotted lines represent carbon hydrogen bond interactions, pink dotted lines represent π–π T-shaped interactions, and the orange dotted line represents a π–sulphur interaction with the residue M986.

On the other hand, DOX that forms hydrophobic interactions with just five residues in the binding pocket (I731, l762, F759, F728, and M986) has a very favorable binding energy of -196.862 kcal/mol. The stability in the binding pocket could be attributed to the presence of hydrogen bonds interactions involving five different residues in the docked pose (Figure 16), three of them are strong conventional type of hydrogen bond, and two are weak carbon type of hydrogen bond. Additionally, DOX may be stabilized by the π–sulphur type of interaction present between the π electron cloud of one of the aromatic rings in the structure and the lone pair of electrons cloud of the sulphur atom in M986. DOX is known to be a substrate of P-gp [51,52] so the docking results obtained are in agreement with the literature available and with the experimental transport assay results reported in Figure 11. 

**Figure 17:**
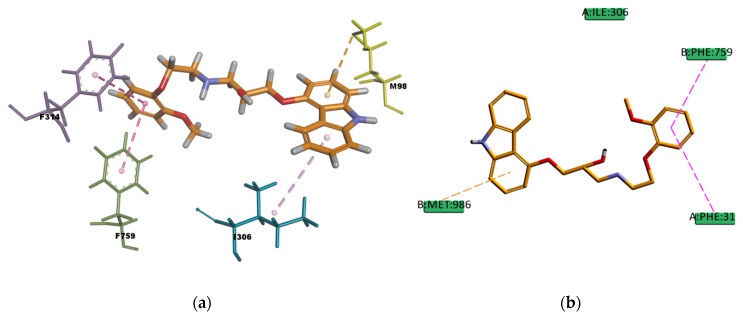
Carvedilol (CAR) top-ranked pose obtained with CDOCKER algorithm. (**a**) 3D view of CAR interactions in the binding pocket. The light-rose dotted lines represent hydrophobic interactions, pink dotted lines represent π–π T-shaped interactions, and the orange dotted line represents a π–sulphur interaction. Residue M986 involved in π–sulphur interaction is highlighted in yellow; (**b**) 2D interaction diagram of CAR with *h*P-gp interacting residues. The pink dotted lines represent π–π T-shaped interactions, and the orange dotted line represents the π–sulphur interaction with the residue M986.

**Figure 18:**
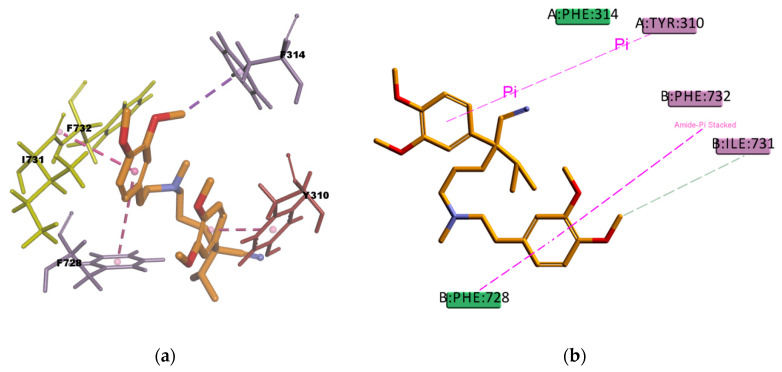
Verapamil (VER) top-ranked pose obtained with CDOCKER algorithm. (**a**) 3D view of VER interactions in the binding pocket. The pink dotted lines represent π–π interactions, and the purple dotted line represents a π–sigma interaction. Residues I731 and F732 involved in the Amide···π stacking interaction are highlighted in yellow; (**b**) 2D interaction diagram of VER with *h*P-gp interacting residues. The pink dotted lines represent π interactions, and the light-green line represents a carbon hydrogen bond interaction with residues I731.

CAR, another well-known substrate of P-gp [50], forms hydrophobic interactions with three residues (I306, F314, and F759) in the binding pocket; these interactions are mainly of π character, two of them are π–π type of interaction and one is π–alkyl type of interaction. The docking pose is also forming one π–sulphur type of interaction (Figure 17) between the π electron cloud of one of the aromatic rings in the carbazol structure and the lone pair of electrons cloud of the sulphur atom in M986; π–sulphur interactions have been well-recognized to play an important role in chemical and biological recognition, as well as in drug development [53,54], thus they may have a big contribution in stabilizing the molecule into the receptor binding site. The binding mode of the VER pose (Figure 18) shows hydrophobic interactions with four residues (Y310, F314, F732, and F728) in the binding pocket, all of them π type of interactions, one π–sigma type of interaction, two π–π type of interaction, and one amide···π stacking interaction, in which the π-surface of the amide bond between residues I731 and F732 stacks against the π-surface of the one aromatic ring in VER. Amide···π stacking interactions are common and significant in protein structures [55] and sometimes they play an important role in ligand binding [56,57]. VER binding mode also involves a weak carbon hydrogen bond interaction with residue I731, which contributes in stabilizing the ligand–protein complex. The estimated binding energies of CAR and VER, shown in Table 14, indicate that the complexes are stable and with a good binding affinity. The docking results are in agreement with the literature available as CAR and VER are well-known substrates of P-gp [44,50].

**Figure 19:**
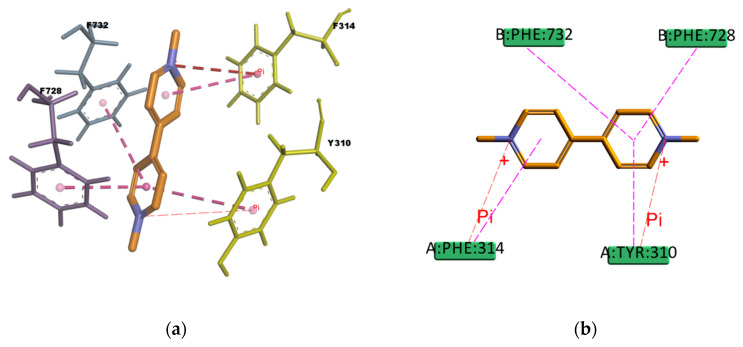
Paraquat (PQ) top-ranked pose obtained with CDOCKER algorithm. (**a**) 3D view of PQ interactions in the binding pocket. The pink dotted lines represent π–π T-shaped interactions, and the red dotted lines represents cation–π interactions. Residues F314 and Y310 involved in the cation–π interactions are highlighted in yellow; (**b**) 2D interaction diagram of PQ with *h*P-gp interacting residues. The pink dotted lines represent π T-shaped interactions, and the red dotted lines represent cation–π interactions with residues F314 and Y310.

**Figure 20:**
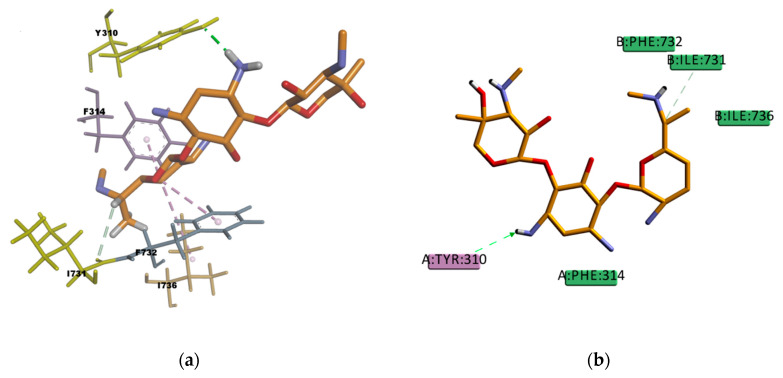
Gentamicin (GEN) top-ranked pose obtained with CDOCKER algorithm. (**a**) 3D view of GEN interactions in the binding pocket. Green dotted line represents a conventional hydrogen bond interaction, light-green dotted line represents a carbon hydrogen bond interaction, and light-rose dotted lines represent hydrophobic interactions. Residues Y310 and I731 involved in hydrogen bond interactions are highlighted in yellow; (**b**) 2D interaction diagram of GEN with *h*P-gp interacting residues. The green dotted line represents a conventional hydrogen bond interaction with residue Y310, and the light-green dotted line represents a carbon hydrogen bond interaction with residue I731.

Regarding PQ and GEN compounds, even though the experimental transport assay results in Figure 11 show that these compounds at 50 µM do not interfere with the P-gp mediated efflux of R123 out of Caco-2 cells, the estimated binding energies have very favorable values. The literature available reveals different results regarding PQ and P-gp interaction (e.g., some authors stated that PQ is transported by P-gp [58] while others stated that it is not a P-gp substrate [59]). In the resulting binding pose (Figure 19), PQ forms hydrophobic interactions involving four residues in the binding pocket (Y310, F314, F728, and 732), all of them π–π type of interaction. It also presents a cation–π interaction between the positively charged nitrogen of PQ and the polarizable π electron cloud of the aromatic ring in F314 and Y310. These are essentially electrostatic interactions due to the negatively charged electron cloud of π systems, and are involved in many drug–receptor interactions, demonstrating that they play an important role in ligand–binding affinity [60]. On the other hand, GEN binding pose (Figure 20) forms hydrophobic interactions with three residues in the binding pocket (I736, F314, and F732), two π–Alkyl type of interactions and one Alkyl type of interaction, besides, it forms one conventional strong type of hydrogen bond with Y310 and one weak carbon type of hydrogen bond with I731, interactions which may explain the stability in the binding pocket reflected by the favorable binding energy. Despite of the binding energies which reflect certain stability in the binding site, both PQ and GEN are hydrophilic compounds, property which may interfere with the capability of both compounds in reaching the binding pocket due to its highly hydrophobic environment. 

**Figure 21:**
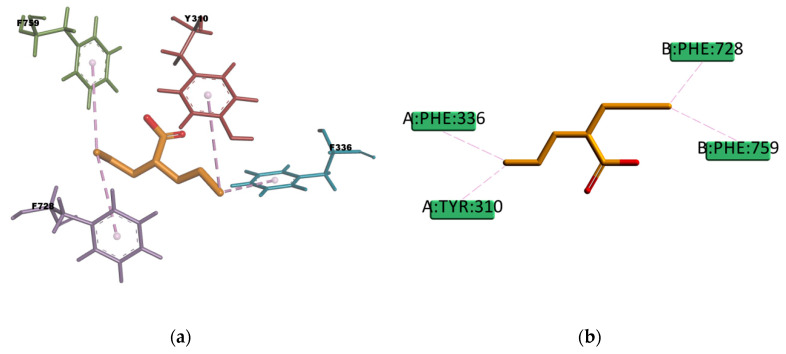
Valproic acid (VPA) top-ranked pose obtained with CDOCKER algorithm. (**a**) 3D view of VPA interactions in the binding pocket. Light-rose dotted lines represent hydrophobic interactions; (**b**) 2D interaction diagram of VPA with *h*P-gp interacting residues. Light-rose dotted lines represent hydrophobic interactions.

**Figure 22:**
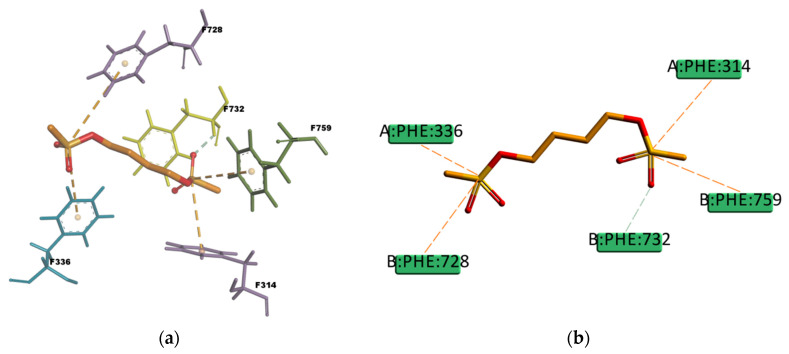
Busulfan (BU) top-ranked pose obtained with CDOCKER algorithm. (**a**) 3D view of BU interactions in the binding pocket. The light-green dotted line represents a carbon hydrogen bond interaction, and the orange dotted lines represent π–sulphur interactions. Residue F732 involved in a carbon hydrogen bond interaction is highlighted in yellow; (**b**) 2D interaction diagram of BU with *h*P-gp interacting residues. The light-green dotted line represents a carbon hydrogen bond interaction, and the orange dotted lines represent π–sulphur interactions with residues F336, F728, F314, and F759.

**Figure 23:**
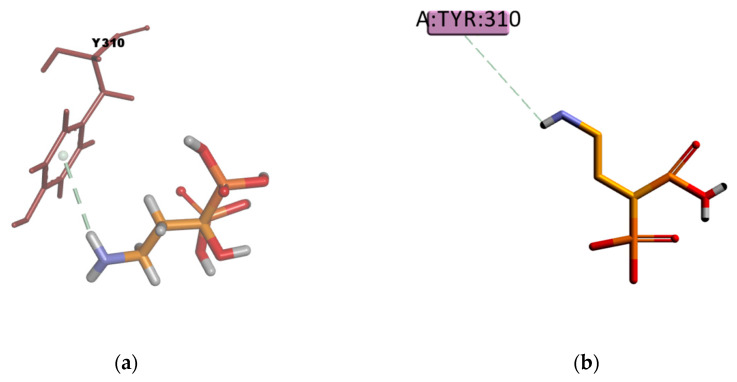
Pamidronate (APD) top-ranked pose obtained with CDOCKER algorithm. (**a**) 3D view of VPA interactions in the binding pocket. The light-green dotted line represents a π–donor hydrogen bond interaction; (**b**) 2D interaction diagram of APD with *h*P-gp interacting residues. The light-green dotted line represents a π–donor hydrogen bond interaction with residue Y310.

VPA and BU compounds resulted with the highest estimated binding energies in the set of docked compounds, as well as not transported by P-gp in the experimental transport assay results (Figure 11). However, looking at the selected docking pose, VPA is forming π–Alkyl type of interactions with four residues in the binding pocket (Y310, F336, F728, and F759) (Figure 21), i.e., interactions which may explain why in some literature articles VPA is reported as a P-gp inducer [61] or a weak affinity inhibitor [62]. On the other hand, the resulting docked pose of BU (Figure 22) involves four π–sulphur type of interactions with residues F336, F728, F314, and F759, and one weak carbon hydrogen bond type of interaction with F732 in the binding mode. In experimental conditions, due to the slightly hydrophilic nature, BU may experience difficulties reaching the binding site, which, as previously mentioned, is located in a highly hydrophobic environment. The APD compound instead, presents just one π–donor hydrogen bond type of interaction between the π electron cloud of the aromatic ring in Y310 and the hydrogen atom of the amine group in APD (Figure 23). This only interaction seems to confer certain stability to the complex according to the calculated binding energy (Table 14), despite that, the hydrophilic nature of APD may certainly interfere in reaching the binding place as the results of the experimental transport assay did not show any interaction between APD and P-gp.

Interestingly, six of the eight known active compounds of P-gp in the docking set (CsA, AM, DIG, DOX, LPM and RMP) involved simultaneous interactions with residues Y307, Y310, F728, and F732 in the binding mode, indicating that these residues may play a crucial role in ligand recognition and binding. These four residues were also found to interact with the inhibitor PBDE (polybrominated diphenyl ether)-100 in the co-crystallized structure of *m*P-gp (PDB ID: 4XWK) demonstrating their relevance in ligand binding and the consistency between the amino acids predicted by molecular docking and the experimental data available. 

Some compounds, such as PQ and GEN might not interact with the P-gp as shown in our study, despite the calculated binding energies that reflect a certain stability in the binding site. Although this could be seen as evidence of the lack of predictability of this approach to identify compounds that interact with P-gp, it should also be noted that these results highlight the importance of the physicochemical properties of the compounds (particularly their lipophilicity) that can prevent them from reaching the binding pocket of P-gp. Moreover, it should be noted that the predictive value of the model applied is still very good for compounds that have been found not to bind to P-gp, since for these compounds the ability to reach the binding pocket of P-gp would not make a difference.

#### 2.3.2. Docking Into the *h*P-gp cryoEM Structure

The set of thirteen compounds was also docked into the experimentally solved cryo–electron microscopy structure of *h*P-gp (PDB ID: 6QEX). The binding region was delineated by those atoms within a radius of 9.5 Å for CDOCKER and GOLD calculations, utilizing the experimental coordinates of the cryoEM *h*P-gp ligand (PDB ID: 6QEX). The selection of the binding site and the settings for the docking calculations were validated via re-docking (ligand reproduction) procedure, obtaining heavy-atoms RMDS values of 1.2723 Å, 1.3208 Å, 1.4630 Å for CDOCKER calculations and 1.0283 Å, 1.1974 Å, 1.2669 Å for GOLD calculations. The RMSD results are in agreement with the accepted threshold of 2 Å (see Table S3 and Figure S5 in Supplementary Materials). 

The resulting poses are distributed within the TM regions of P-gp (Figure S2) and show interactions with protein residues of several TM helices located throughout the binding region (3D diagrams of the obtained binding poses can be found in Supplementary Materials Figure S3). The interactions of the ligands in the binding pocket in the homology model compared to those in the experimentally determined structure (Table 15) are in good agreement. The types of interactions for most compounds are equivalent in both docking studies and share many of the interacting amino acid residues; e.g., CsA shows mainly interactions of hydrophobic character with residues in the binding pocket, a large number of π interactions, such as π–alkyl interactions in the presence of hydrogen bonds, which gives the complex a high stability. The stability of the complex is also reflected in the calculated binding energy values given in Table 16. The only compound in the set that had no interacting amino acids in common with the homology model was PQ. Nevertheless, the nature of the interactions agrees well with the results in the homology model, π–cation and the π–π interactions are involved in the stabilization of the complex. The calculated binding energy values are also comparable with respect to the binding stability of the complexes, although the absolute values are about half of the energy values reported in the homology model, e.g., the most stable complex in the set of docked compounds is CsA both in the homology model and in the cryoEM structure of *h*P-gp; the less stable complexes are VPA and BU in both docking systems as well.

## 3. Materials and Methods 

### 3.1. Protein Homology Modeling

The 3D protein homology model of *h*P-gp was constructed using three different tools, SWISS-MODEL [25], I-TASSER [22,23,24] and Discovery Studio 4.1/Modeler 9.12 [20,21]. The complete *h*P-gp protein sequence, which consists of 1280 amino acids, was retrieved from the UniProtKB database (accession number P08183).

#### 3.1.1. Template Selection and Alignment

The selection of the templates was based on sequence similarity with known protein structures (homologous) from the protein data bank (PDB) repository. SWISS-MODEL and Discovery Studio 4.1 protocols identified suitable templates based on BLAST [63] while I-TASSER identified structure templates by using LOMETS [64,65]. 

The target and template sequences were aligned in order to analyse the sequence conservation. Insertions and deletions were done so that the best alignment could be obtained. Generally, it is preferable to include more than one template in the alignment because it could provide a better fitting of regions where the percentage of identity is very low with the use of a single template. 

#### 3.1.2. Model Generation

The overall structure of the *h*P-gp (based on full-length sequence as retrieved from UniProtKB accession number P08183) was modeled including the nucleotide binding domains (NBDs) and the flexible linker region. Since the quality of the constructed *h*P-gp model is directly dependent on the quality of the template used, the full primary sequence and secondary structure informations related to the *h*P-gp were utilized, including NBDs. Moreover, it seems that the linker region is important for the stabilization of the NBDs, where it acts as a ‘damper’ reducing the movements of the cytoplasmic regions of P-gp [66]; therefore, it was included in the model as well.

In general, the steps for generating a protein homology model involve the creation of the target backbone by copying the coordinates of the template-backbone to the target. When the residues are identical also the protein side-chain coordinates are copied. The gaps in alignment due to insertions and deletions are modeled by loop modeling. The side- chains can be built by searching every possible conformation for every torsion angle of the side-chain and selecting the one that has the lowest interaction energy with neighbouring atoms. A rotamer library can be also used for this purpose, which has all the favorable side-chain torsion angles extracted from known protein crystal structures. Finally, the geometry of the resulting model is minimized by using a force field.

The different tools employed here in the modeling of *h*P-gp differ in the algorithms used for building the model and in the methods utilized for the model refinement. 

#### SWISS-MODEL

This tool extracts the initial structural information from the template structure. Insertions and deletions are resolved looking for viable candidates in a structural database. Final candidates are selected using statistical potentials of mean force scoring methods. If no candidates can be found, then a conformational space search is performed using Monte Carlo conformational techniques. Non-conserved side-chains are modeled using an in-house backbone-dependent rotamer library. The optimal configuration of rotamers is estimated using the graph-based TreePack algorithm [67] by minimising the SCWRL4 energy function [68]. As a final step, small structural distortions, unfavorable interactions or clashes introduced during the modeling process are resolved by energy minimisation. SWISS-MODEL uses CHARMM27 force field for parameterisation.

SWISS-MODEL assesses the quality of the model through the GMQE (Global model quality estimation) and the QMEAN [26] score. The GMQE has values between 0 and 1, reflecting the accuracy of the model built with that specific alignment and template. The higher the number, the higher the reliability of the model is. The QMEAN score indicates the degree of nativeness of the structure in the model. Values around 0 mean good quality agreement between the modeled structure and experimental structures of similar size. Values less than −4 indicate models of low quality. In addition to the previous scores, the expected similarity to the native structure for each residue in the model can be checked through the Local Quality plot. Usually, residues showing a score below 0.6 are expected to be of low quality.

#### I-TASSER

I-TASSER tool uses fragments excised from the PDB templates, reassembles them into full-length models by using Monte Carlo simulations, and builds the loops by ab initio modeling. The large ensemble of structural conformations, called decoys, is then clustered by SPICKER [69] program based on the pair-wise structure similarity. The final full-atomic models are obtained by REMO [70], which builds the atomic details from the selected I-TASSER decoys through the optimization of the hydrogen-bonding network and for this purpose, it utilizes the CHARMM22 force field parameters. Five models are reported, which correspond to the five largest structure clusters. 

For assessing the global accuracy of the model I-TASSER employs the C-Score, the TM-Score [27], and the RMSD. The C-Score is a confidence score calculated based on the significance of the threading templates alignments and the convergence parameters of the structure assembly simulations. It has values between −5 and 2, where higher values indicate a model with a high confidence and vice versa. The TM-Score and the RMSD are predicted based on the C-Score as they are highly correlated. The correlation coefficient of C-score of the first model with TM-score and RMSD are 0.91 and 0.75, respectively. The TM-Score is a measure of the structural similarity between the predicted model and the native structure; unlike RMSD, it is insensitive to the local modeling error because it weights the small distance stronger than the large one; for RMSD a local error will arise a big RMSD value, even though the global topology is correct. TM-score has values that between 0 and 1, i.e., values greater than 0.5 indicate a correct topology of the model while values less than 0.17 indicate random similarity. The local accuracy of the model can be visualized in the Estimated Local Accuracy Plot, which shows the distance deviation between the residue positions in the model and the estimated native structure.

#### Discovery Studio 4.1/Modeler 9.12

Modeler uses restraints on the spatial structure of the amino acid sequence and ligands to be modeled. The output is a 3D structure that satisfies these restraints as much as possible. The program automatically derives the restraints only from the known related structures and their alignment with the target sequence. The restraints can be on distances, angles, dihedral angles, pairs of dihedral angles and some other spatial features. During the model refinement, conjugate gradient and simulated annealing molecular dynamics (MDs) are used to optimize the positions of heavy atoms. Modeler utilizes the CHARMM22 force field for parameterisation.

Discovery studio uses the Verify Score for assessing the validity of the modeled 3D structure, which measures the compatibility of each residue in the current 3D environment. As a reference point the Verify Expected High and Low Score for a protein of the same size are given. If the calculated Verify Score is greater than the Expected High Score, the structure is likely to be correct. Conversely, if it is lower than the Expected Low Score then the structure is almost certainly misfolded. In general, the closest the Verify Score is to the Verify Expected High Score value, the better the quality of the model is. Discovery Studio also reports the Probability Density Function (PDF) Energy and the Discrete Optimized Protein Energy (DOPE) [71] scores. The lower these values are the better the model is. The local accuracy of the model can be visualized in the Verify Score Plot, which gives the compatibility score of each residue in the given 3D structure. 

#### 3.1.3. Assessment of the Models

The validation of the models developed was performed using the PROCHECK [28], VERIFY 3D [29], ERRAT [30], and PROVE [31] programs, which are available at the Structural Analysis and Verification Server (SAVES) [72]. 

PROCHECK was used for assessing the stereochemical quality of the protein structure. It checks the protein backbone conformation by analysing the torsion angles phi (φ) and psi (ψ) of the amino acid residues in the modeled protein utilizing the Ramachandran plot. 

VERIFY 3D program analyses the compatibility of the assembled atomic model (3D) with its corresponding amino acid primary sequence (1D). It classifies each residue in the protein into one of the 18 classes according to the residue’s structural environment in the input model. The propensity of each amino acid to exist in each structural environment class is calculated according to statistics collected from structures in the PDB repository. The final score given to the protein structure is the sum of propensities of the individual residues. If at least 80% of the amino acids have a score greater than or equal to 0.2 in the 3D/1D profile then the test is considered passed.

ERRAT is an algorithm that analyses the statistics of non-bonded interactions between different atom types (CC, CN, CO, NN, NO, and OO). The ERRAT score is expressed as the percentage of the protein for which the calculated error value falls below the 95% rejection limit. Good high-resolution structures generally produce values around 95% or higher. For lower resolutions (2.5–3.0 Å) the average overall quality factor is around 91%. The generally accepted ERRAT score for considering a good quality model is of at least 50% of the structure below the 95% confidence limit. This is a very useful tool for assessing the reliability of a model. 

PROVE calculates the volumes of atoms in macromolecules using an algorithm which treats the atoms like hard spheres and calculates a statistical Z-score deviation of the model from highly resolved and refined PDB deposited structures. If the percentage of buried atoms in the structure is less than 1% the test is considered passed, otherwise if it is between 1% and 5% then a warning is given on the structure. When the percentage of buried atoms is greater than 5%, it is considered that there are some errors present in the structure. 

### 3.2. Molecular Docking Calculations 

The molecular docking calculations were carried out on a set of thirteen compounds, among them eight well-known molecules which interact with P-gp as substrates, inhibitors or both: CsA, a high affinity substrate [43] and inhibitor [73] of P-gp, AM, a known substrate [50] of P-gp, DOX, a substrate of P-gp [51,52], DIG, a high affinity P-gp substrate [48], LPM a known substrate [49] of P-gp, RMP, a substrate [46] and inducer [47] of P-gp, VER, a well-known substrate of P-gp, CAR, a well-known substrate of P-gp [50], and five non-interacting compounds with P-gp: VPA, BU, GEN, APD, and PQ [59]. We used the available knowledge on the interaction of substrates and inhibitors with P-gp to elucidate if the molecular docking is able to differentiate between active and non-active P-gp compounds, and for determining to what extent the amino acids predicted by molecular docking are consistent with experimental data available. 

The ligand–P-gp complexes were built by docking the ligand into the homology model of *h*P-gp and the cryo–EM structure of *h*P-gp (PDB ID: 6QEX) using the Dock Ligands protocol inside Discovery Studio 4.1 Client [20]. Two algorithms, CDOCKER [36] and GOLD [37,38], where utilized to investigate the binding affinities and conformations on a set of thirteen compounds, including some well-known compounds that interact with P-gp as substrates, inhibitors, or both. The docking studies were performed by not taking into consideration the flexible linker region present in the *h*P-gp structure. This region is more than 30 Å away from the binding pocket, therefore it does not appear to be involved in drug binding.

The selection of the binding site and the settings for the molecular docking simulations using the constructed homology model and the cryoEM structure of *h*P-gp were validated *via* the procedure called “self-docking” or “re-docking”. Re-docking procedure is a widely used method for the validation of all docking settings done prior to perform molecular docking calculations. The quality of all performed structure-based (molecular docking) settings was initially verified by docking the co-crystallized ligand PBDE-100 and cryoEM taxol into their defined binding pockets [74] and comparing them with their experimental conformation, i.e., the reproduction of their spatial conformation and orientation (Tables S2 and S3, Figures S4 and S5). The experimental coordinates of PBDE-100 and taxol as well as surrounding amino acid residues were used to define the binding cavity, for our model and the cryoEM *h*P-gp separately. During the re-docking validation procedure, PBDE-100 (our *h*P-gp model) and taxol (cryoEM *h*P-gp) were first removed from their binding site and re-docked three times. As a evaluation criteria for a successfully performed re-docking validation, the heavy-atoms RMSD values (RMSD ≤ 2 Å) between each re-docking obtained poses and natively present ligand (PBDE-100 and taxol, respectively) were calculated [75]. 

After proper validation, each ligand under investigation was docked up to ten times with the same docking parameters obtained by the re-docking validation, while the quality of the obtained docking poses was quantified by –CDOCKER Energy and GoldScore fitness function, i.e. the main scoring functions of CDOCKER and GOLD, respectively. The resulting docking poses were subsequently rescored with fourteen scoring functions implemented in Discovery Studio 4.1 Client. A total of two docking runs were performed, each of which scored with 15 different fitness functions.

The following general steps were followed for the docking simulations:

1. Ligand preparation: The CHARMm force field from the simulation tool was applied to the ligands and a minimization protocol was performed
2. Protein preparation: The CHARMm force field from the simulation tool was applied to the target protein, a minimization protocol was performed and the binding site based on ligand was defined.
3. Running CDOCKER or GOLD protocol.
4. Scoring of docked Ligand Poses.
5. Calculation of ligands Binding Energies.

#### 3.2.1. Docking with CDOCKER

CDOCKER is a grid-based molecular docking method that employs CHARMm force field to dock ligands into a receptor binding site. The receptor is held rigid, while the ligands are allowed to be flexible during the refinement. Random ligand conformations are generated from the initial ligand structure through high temperature molecular dynamics, followed by random rotations. When these conformations are translated to the active site, the candidate poses are refined by grid-based (GRID1) simulated annealing, and a final grid-based or full force field minimization. CDOCKER uses soft core potentials, which are found to be effective in exploring conformational space of small organics and macromolecules. The non-bonded interactions which involve van der Waals (vdW) and electrostatics are softened at different levels, except during the final minimization step [36]. 

Ten conformations for each ligand were generated in the binding site of the *h*P-gp, which was created as a spherical region defined by those atoms within a radius of 15 Å from the co-crystallized ligand originating from *m*P-gp (PDB ID: 4XWK). The selection of the binding site was validated by the re-docking procedure. Random conformations were generated using specific molecular dynamics steps while the system is heated to 700 K in 2000 steps, then cooled to 300 K in 5000 steps. The final refinement step of minimization was performed using full potential. The minimized docking poses were then clustered, based on a heavy atom RMSD approach. The final ranking was based on the total docking energy, which is composed of the ligand’s intramolecular energy and the ligand receptor interaction.

#### 3.2.2. Docking with GOLD

GOLD (Genetic Optimization for Ligand Docking) uses a genetic algorithm to explore the full range of ligand conformational flexibility with partial flexibility of the protein active site while searching for favorable ligand poses. A population of chromosomes is manipulated during a genetic algorithm run, with each chromosome representing a trial docking. A chromosome contains all the information needed to completely define a trial ligand pose and is associated with a fitness value computed from the scoring function. Different values of the genetic algorithm parameters may be used to control the balance between the speed of GOLD and the reliability of its predictions.

Different conformations for each ligand were generated in the binding site of the *h*P-gp, which was created as a spherical region defined by those atoms within a radius of 24.7 Å from the ligand co-crystallized in the structure of the *m*P-gp PDB ID: 4XWK. The radius of the sphere is significantly higher than in the case of CDOCKER, because some of the residues in the binding site are allowed to move. The selection of the binding site was validated by the re-docking procedure. Flexible docking was performed, meaning that the side chains of some amino acids in the binding site were able to rotate continuously about single bonds during the docking simulation. Ten residues in the binding pocket were selected to be flexible based on some drug binding residues reported experimentally in references [19,76]. The selected residues were F303, Y307, Y310, F314, Q725, F728, F732, F759, F983, and Q990 with hydrophobic, aromatic and polar characteristics.

#### 3.2.3. Scoring of Docked Ligand Poses and Calculation of Binding energies

The docked ligand poses obtained from each algorithm were re-scored with various scoring functions through the Score Ligand Poses protocol in Discovery Studio 4.1 Client. Then, the sum of ranking differences (SRD) methodology [77] was employed for the comparison of the performance of the scoring functions calculated. The SRD is a robust statistical method, specifically developed for method comparison tasks. It evaluates Manhattan distances of a set of rank transformed vectors, in this case, the different fitness functions, from a reference vector which corresponds to a hypothetical ideal reference method. The reference vector can be a “gold standard” or experimental values, where available, or a consensus method based on data fusion. In this case, we have defined the reference vector as the average value of the scoring functions. 

For the selection of the top-ranked pose of each docked ligand, a consensus ranking scheme was employed. Instead of combining the raw scoring values coming from different scoring functions, the ranks produced by these scoring functions were combined in the following manner: first, the rank derived from each scoring function was produced. Then, for a specific combination of scoring functions, a fused rank was computed as the geometric mean [78] of the compound’s rank in the individual models. The scoring functions selected to be combined were those that showed a better performance in the SRD results.

The average binding energy across the set of related poses for each ligand was calculated. The binding free energies between the *h*P-gp 3D model and the set of docked ligand poses obtained were estimated using the Calculate Binding Energies protocol in Discovery Studio 4.1, in which the free energy of binding for a receptor–ligand complex is calculated from the free energies of the complex, the receptor, and the ligand according to Equation (1). The protocol uses CHARMm based energies and implicit solvation methods to estimate these free energies and thus calculate an estimate for the overall binding free energy.

Energy_Binding_ = Energy_Complex_ − Energy_Ligand_ − Energy_Receptor_ (1)

### 3.3. Caco-2 Pump Out Assay

The interaction with the efflux pump P-gp of nine compounds present in the docking set (CsA, AM, DOX, VER, VPA, BU, GEN, ADP, PQ) was characterized using a new protocol based on the use of Caco-2 cells directly seeded on 96-well plates and the use of fluorescent substrates for efflux pumps, Rhodamine-123 (R123) for the P-gp case. The experiment was performed as described in reference [45]. 

Shortly, Caco-2 cells were washed with HEPES-buffered Ringer’s (RH) solution (NaCl 150 mM, KCl 5.2 mM, CaCl2 2.2 mM, MgCl2 0.2 mM, NaHCO3 6 mM, Glucose 2.8 mM, HEPES 5 mM, water for injection), pH = 7.4 and incubated for 120 min with 10 μM R123. After incubation, cells were washed with RH and incubated with test compounds for one hour during which the rate out (Kout) of R123 (λex = 485 nm and λem = 538 nm) was monitored every 2 minutes with a microplate fluorescence reader (Fluoroskan Ascent FL, Thermo Labsystems, Issy-Les-Moulineaux, France) at 37 °C, after which the cell viability was assessed using MTT cytotoxicity assay kit. Rhodamine Kout was calculated as the slope of the curve of the cumulative R123 fluorescence against the time. Verapamil was used as a positive control and diazepam as negative control.

Amiodarone, busulfan, cerium dioxide nanoparticles, cyclosporin A, diazepam, gentamicin sulfate, lead (II) chloride, paraquat dichloride, rhodamine 123, valproic acid and verapamil were obtained from Sigma-Aldrich (Saint Quentin Fallavier, France); doxorubicin hydrochloride was obtained from J&K Scientific (Lommel, Belgium) and pamidronate was obtained from Tebu-bio (Heerhugowaard, The Netherlands).

Cerium dioxide, doxorubicin, gentamicin C, lead (II) chloride, pamidronate and paraquat dichloride were dissolved in water, cyclosporin A, valproic acid and verapamil were dissolved in DMSO, amiodarone was dissolved in methanol and busulfan in acetone.

MTT ((3-(4,5-dimethylthiazol-2-yl)-2,5-diphenyltetrazolium bromide) tetrazolium reduction assay), Cell Proliferation, and Cytotoxicity Assay Kit were obtained from Alphabioregen (Boston, MA, USA).

## 4. Conclusions

The quality assessment of the *h*P-gp models developed suggested that the overall folding of the 3D structure is as good as the available crystal structure of the *m*P-gp (PDB ID: 4M1M) and therefore, reliable and suitable for further in silico structure-based studies. The employed method was capable of generating an *h*P-gp model similar to the near-native *m*P-gp. The characterization of the binding pocket of our homology model resulted in a big hydrophobic surface area. These results are in agreement with biochemical investigations, which concluded that the drug-binding pocket is assembled mostly of hydrophobic residues, creating a lipophilic environment. In silico QSAR models, classifying between active and non-active compounds, generally include physicochemical descriptors measuring lipophilicity of the molecules such as logP or log D (pH 7.4), which is in good correlation with our observations.

The analysis of the binding poses suggests that the large number of π interactions together with the simultaneous presence of hydrogen bond interactions contribute to the stability of the ligand–protein complex in the binding site; the hydrogen bond interactions present are mostly of weak character (carbon hydrogen bonds type C–H…O) with a greater dispersive component. Identical interacting amino acid residues observed in the *m*P-gp crystal structure (PDB ID: 4XWK) and the *h*P-gp cryoEM structure (PDB ID: 6QEX) contribute to drug binding in the *h*P-gp homology model, e.g., Q725, Y307, F983, which occur in both the *m*P-gp crystal structure and the *h*P-gp cryoEM structure.

Some amino acid residues that contribute to drug binding in our *h*P-gp homology model are also present in one or the other of the two experimentally solved structures, e.g. the amino acid residues Q990, A987, I340, M986, F343 are also interacting residues in the *h*P-gp cryoEM structure, while the residues Y310, F314, F728, F732, F759 are interacting residues in the *m*P-gp crystal structure, which demonstrates the consistency between the interacting amino acid residues predicted by molecular docking calculations and the co-crystallized data available. 

Examination of the ligand–*h*P-gp complexes provides a considerable insight into the drug binding mode for the set of investigated ligands. It was able to reveal different interacting modes for different classes of compounds; thus, molecular docking studies should not be underestimated in this field. In some cases, the experimental data show a great amount of controversy, therefore the combination of computational methods and experimental data from efflux pump transport assays is essential in the complex field of P-glycoprotein. 

## Figures and Tables

**Table 1:** QMEAN and GMQE scores of the models generated by the SWISS-MODEL tool.

	Model 1	Model 2	Model 3	Model 4	Model 5
QMEAN	−2.71	−2.80	−2.72	−3.13	−3.28
GMQE	0.87	0.86	0.81	0.80	0.79

**Table 2:** C-score, TM-Score, and root mean square deviation (RMSD) of the models generated by the I-TASSER tool.

	Model 1	Model 2	Model 3	Model 4	Model 5
C-Score	0.49	−1.35	−1.24	0.29	−2.30
TM-score	0.78 ± 0.10				
RMSD	8.3 ± 4.5				
Number of decoys	2702	527	512	1993	0.82
Cluster density	0.1620	0.0256	0.0286	0.1325	0.0099

**Table 3:** Probability Density Function (PDF) Total Energy and DOPE Score of the models generated with Discovery Studio 4.1 Client/Modeler 9.12 tool.

	PDF Total Energy	DOPE Score
Model 1	80,529.9	−151.500
Model 2	81,619.5	−151.832
Model 3	79,999.4	−153.656
Model 4	79,720.3	−153.494
Model 5	80,422.7	−153.242
Model 6	80,065.3	−153.370
Model 7	81,341.2	−151.484
Model 8	79,800.9	−153.459
Model 9	80,116.5	−153.266
Model 10	80,489.5	−153.900
Model 11	81,588.3	−152.353
Model 12	80,908.7	−152.955
Model13	80,406.8	−153.855
Model 14	80,046.3	−153.624
Model 15	80,453.5	−153.758
Model 16	79,976.6	−154.280
Model 17	80,841.3	−153.293
Model 18	82,785.2	−151.571
Model 19	79,876.5	−153.904
Model 20	80,607.2	−152.350

**Table 4:** Verify Scores of the Model 4 generated by Discovery Studio 4.1 Client/Modeler 9.12 tool.

	Verify Score	Verify Expected High Score	Verify Expected Low Score
**Model 16**	513,011	593,427	267,042

**Table 5:** Ramachandran Plot Statistics for the *h*P-gp models and the crystal structure of *m*P-gp (PDB: 4M1M)

	4M1M	Model SM ^1^	Model IT ^2^	Model DS ^3^
Residues in most favored regions	91.5%	92.0%	87.7%	92.0%
Residues in additional allowed regions	7.0%	5.5%	10.0%	6.3%
Residues in generously allowed regions	1.3%	1.7%	1.4%	1.1%
Residues in disallowed regions	0.1%	0.8%	0.9%	0.6%

^1^ SWISS-MODEL. ^2^ I-TASSER. ^3^ Discovery Studio.

**Table 6:** Alignment of the models selected from each modeling tool with respect to the crystal structure of *m*P-gp (PDB: 4M1M). Main-chain RMSD (in angstrom) are below the diagonal and Number of Overlapping Residues above the diagonal.

	4M1M	Model SM ^1^	Model IT ^2^	Model DS ^3^
4M1M		1181	1167	517
Model SM ^1^	0.2430		1167	517
Model IT ^2^	0.7000	0.7010		517
Model DS ^3^	1.8330	1.8350	1.8680	

^1^ SWISS-MODEL. ^2^ I-TASSER. ^3^ Discovery Studio.

**Table 7:** RMSD in angstrom of the models selected from each modeling tool with respect to the crystal structure of *m*P-gp (PDB: 4M1M).

	Reference	C-Alpha	Side-Chain	All Protein
Model SM ^1^	4M1M	0.173	0.473	0.369
Model IT ^2^	4M1M	0.528	2.074	1.510
Model DS ^3^	4M1M	1.809	2.365	2.098

^1^ SWISS-MODEL. ^2^ I-TASSER. ^3^ Discovery Studio.

**Table 8:** Verify 3D, ERRAT and PROVE Scores of the models selected from each modeling tool.

	Verify 3D	ERRAT	PROVE
Model SM ^1^	44.69%	94.0120	6.8%
Model IT ^2^	63.41%	96.0884	5.6%
Model DS ^3^	45.22%	80.5934	7.2%
PDB ID: 4M1M	65.20%	86.5620	0.0%

^1^ SWISS-MODEL. ^2^ I-TASSER. ^3^ Discovery Studio.

**Table 9:** Docking runs performed and Scoring functions.

Docking Run	Main Scoring Function	Rescoring Functions
CDOCKER	-CDocker Energy	LigScore2_Dreiding, LigScore1_Dreiding, PLP1, PLP2, Jain, Ludi_1, PMF, PMF04, Goldscore, Chemscore, ChemASP ChemPLP, -Cdocker_IE ^2^, -Cdocker_Eopt ^3^, -Cdocker_IEOpt ^4^
GOLD	GoldScore	LigScore2_Dreiding, LigScore1_Dreiding, PLP, PLP2, Jain, Ludi_1, PMF, PMF04, Chemscore, ChemASP, ChemPLP, -Cdocker_E ^1^, -Cdocker_IE ^2^, -Cdocker_Eopt ^3^, -Cdocker_IEOpt ^4^

^1^ -CDocker energy. ^2^ -CDocker interaction energy. ^3^ -CDocker energy optimized. ^4^ -CDocker interaction energy optimized.

**Table 10:** Fusing ranking scheme.

Docking Run	Fused Scoring Functions Ranks	Fusion OPERATOR
CDOCKER	-Cdocker_IE ^1^, -Cdocker_Eopt ^2^, Chemscore, Goldscore ChemPLP, -Cdocker_IEOpt ^3^	Geometric Mean
GOLD	-Cdocker_IEOpt ^3^, PMF, PMF04, PLP1, PLP2, Goldscore,	Geometric Mean

^1^ -CDocker interaction energy. ^2^ -CDocker energy optimized. ^3^ -CDocker interaction energy optimized.

**Table 11:** SRD ranking of the 16 fitness functions used in the CDOCKER run.

Name	SRD	p% *x* < SRD ≥ *x*	
-Cdocker_Eopt	2360	2.91 × 10^−15^	3.00 × 10^−15^
-Cdocker_IEOpt	2378	4.57 × 10^−15^	4.66 × 10^−15^
ChemScore	3046	1.40 × 10^−7^	1.47 × 10^−7^
-Cdocker_IE	3696	1.80 × 10^−2^	1.83 × 10^−2^
ChemPLP	3764	4.41 × 10^−2^	4.48 × 10^−2^
Goldscore	3920	0.29	0.30
Ludi_1	4022	0.83	0.84
PMF	4034	0.91	0.92
XX1 ^1^	4228	4.99	5.03
ChemASP	4404	15.48	15.57
PMF04	4412	16.22	16.31
Jain	4455	20.21	20.31
-Cdocker_E	4466	21.33	21.45
Q1 ^2^	4497	24.99	25.10
Med ^3^	4685	49.90	50.05
LigScore2_Dreiding	4850	72.29	72.41
PLP2	4862	73.73	73.85
Q3 ^4^	4871	74.94	75.06
PLP1	5134	94.66	94.70
XX19 ^5^	5142	94.97	95.01
LigScore1_Dreiding	5338	99.06	99.07

^1^ First icosaile 5%. ^2^ First quartile. ^3^ Median. ^4^ Last quartile. ^5^ Last icosaile 95%.

**Table 12:** SRD ranking of the 16 fitness functions used in the GOLD run.

Name	SRD	p% *x* < SRD ≥ *x*	
-Cdocker_IEOpt	3526	2.60 × 10^−10^	2.64 × 10^−10^
PMF	3698	9.53 × 10^−9^	9.70 × 10^−9^
PLP2	3850	1.71 × 10^−7^	1.76 × 10^−7^
PMF04	3942	9.50 × 10^−7^	9.72 × 10^−7^
PLP1	4028	4.23 × 10^−6^	4.33 × 10^−6^
Goldscore	4152	3.21 × 10^−5^	3.25 × 10^−5^
-Cdocker_Eopt	4422	1.66 × 10^−3^	1.67 × 10^−3^
ChemPLP	5010	1.00	1.01
XX1 ^1^	5234	4.97	5.01
-Cdocker_IE	5290	7.12	7.16
Chemscore	5320	8.36	8.41
ChemASP	5462	17.48	17.55
Q1 ^2^	5546	24.95	25.04
Ludi_1	5748	47.85	47.97
Med ^3^	5765	49.93	50.06
-Cdocker_E	5853	60.66	60.77
Q3 ^4^	5983	74.98	75.08
LigScore2_Dreiding	6249	93.20	93.25
XX19 ^5^	6298	94.98	95.01
LigScore1_Dreiding	6428	97.96	97.97
Jain	6835	99.95	99.95

^1^ First icosaile 5%. ^2^ First quartile. ^3^ Median. ^4^ Last quartile. ^5^ Last icosaile 95%.

**Table 13:** Estimate of the overall binding free energies of some well-known substrates and inhibitors of P-gp using the homology model.

Name	CDOCKER Binding Energy (kcal/mol)	GOLD Binding Energy (kcal/mol)
CsA ^1^	−268.516	−299.839
AM ^2^	−97.4066	−103.782
DOX ^3^	−196.862	−199.953
DIG ^4^	−211.755	−226.553
LPM ^5^	−106.862	−120.356
RMP ^6^	−214.872	−208.965
VER ^7^	−101.617	−116.922
CAR ^8^	−115.509	−120.018
VPA ^9^	−45.9908	−54.5415
BU ^10^	−59.7798	−69.8159
GEN ^11^	−180.391	−189.158
APD ^12^	−93.428	−99.6753
PQ ^13^	−193.24	−206.54

^1^ Cyclosporine A; ^2^ amiodarone; ^3^ doxorubicin; ^4^ digoxin; ^5^ loperamide; ^6^ rifampin; ^7^ verapamil; ^8^ carvedilol; ^9^ valproic acid; ^10^ busulfan; ^11^ gentamicin; ^12^ pamidronate; ^13^ paraquat.

**Table 14:** Ligand–P-gp interaction types and amino acid residues involved in the binding of some well-known substrates and inhibitors of P-gp using the homology model. The numbers in parenthesis indicate the number of interactions in which the residue is involved.

Name	Hydrogen Bond	Alkyl	π–Sigma	π–Alkyl	π–π	π–Sulphur	Others
CsA ^1^	Q990, Q725, F728	A987, M986 (2), L339, I340 (2), L332, I731, L762, I735 (2), I736	F335	Y307 (2), Y310, F728 (4), F732 (5), F314 (3), F335, F336 (5), F343 (2), F759 (3), F978, F983 (2)	-	-	-
AM ^2^	I731	I731, I735 (2), I736	Y310	Y307, F728 (2), F314 (2) F759 (3)	F983 (2), F732, F728	-	-
DOX ^3^	Y310, Y307, F732, F759, Q990	I731, l762	-	F759	F728 (3)	M986	-
DIG ^4^	F728 (2), Q725, Q990	A987	Y310	Y307, Y310, F728 (3), F732, F336 (2), F343, F983 (2)	-	-	-
LPM ^5^	F728	L762	F732	Y307, Y310, F728 (2), F759, L339, I340	F314	-	-
RMP ^6^	F732, F728, F759	I340, M986	Y310, F732	Y307, Y310 (2), F335, F336, F343, F759 (2), F983 (2) F728, F732	F983	-	-
VER ^7^	I731	-	F314	-	Y310, F728	-	F732 *
CAR ^8^	-	I306	-	-	F314, F759	M986	-
VPA ^9^	-	-	-	Y310, F336, F728, F759	-	-	-
BU ^10^	F732	-	-	-	-	F336, F728, F314, F759	-
GEN ^11^	Y310, I731	I736	-	F314, F732	-	-	-
APD ^12^	Y310	-	-	-	-	-	-
PQ ^13^	-	-	-	-	Y310, F314, F728, F732	-	F314 **, Y310 **

^1^ Cyclosporine A; ^2^ amiodarone; ^3^ doxorubicin; ^4^ digoxin; ^5^ loperamide; ^6^ rifampin; ^7^ verapamil; ^8^ carvedilol; ^9^ valproic acid; ^10^ busulfan; ^11^ gentamicin; ^12^ pamidronate; ^13^ paraquat; * amide···π stacking interaction; ** cation–π interaction.

**Table 15:** Ligand–P-gp interaction types and amino acid residues involved in the binding of some well-known substrates and inhibitors of P-gp using the experimentally solved cryoEM structure of *h*P-gp (PDB ID: 6QEX). The numbers in parenthesis indicate the number of interactions in which the residue is involved.

Name	Hydrogen Bond	Alkyl	π–Sigma	π–Alkyl	π–π	π–Sulphur	Others
CsA ^1^	Q725, Q990 (2), A987, Q347	A987, M876, I340 (2), M986 (2), M69 (2), V991, I306 (2)	W232, F336	H61, W232(2), F303, Y307, Y310 (2), F336 (3), F343 (2), F728 (2), F732, F983 (2)	-	-	-
AM ^2^	-	M986	-	W232, Y307, Y310, F343, F728, M986, L65,	-	M986, M949	M986 *, Q990 *
DOX ^3^	Y310, Q990 (2), M986, Q347, A871 (2)	L339	-	-	-	M986 (2)	M986 **
DIG ^4^	S344, Q990, I340, A871, G872, F983	A871, L65, M986	-	F336, F728, F983	-	-	F732 ^§§^
LPM ^5^	F303	L339, I340	-	I340, M986	F303	-	-
RMP ^6^	Y307, Y310, Q725, A987, M986, W232	M986, I340 (2)	F728	W232, F303	F728, F983	M986	M986 **
VER ^7^	Y310, Q990, Y307	M986	F728, Y310, F732	-	F336	-	-
CAR ^8^	Y310, F759 (2), F732	-	-	I731, L762	F983(2), F728	M986	-
VPA ^9^	Q990	M986	-	F728, F983	-	-	-
BU ^10^	Y310	-	-	-	-	F728 (2), Y310	F759
GEN ^11^	Y310, 875 (3)	-	-	F728, F983	-	-	-
APD ^12^	Y310, Q724	-	-	-	-	-	-
PQ ^13^	E875	-	-	M986 (2)	F983	-	F983 ^§^

^1^ Cyclosporine A; ^2^ amiodarone; ^3^ doxorubicin; ^4^ digoxin; ^5^ loperamide; ^6^ rifampin; ^7^ verapamil; ^8^ carvedilol; ^9^ valproic acid; ^10^ busulfan; ^11^ gentamicin; ^12^ pamidronate; ^13^ paraquat. Residues in bold are the shared residues in both docking systems. * Halogen interaction; ** sulphur-X interaction; ^§^ cation–π interaction; ^§§^ π–lone pair interaction. The bold printed amino acid residues identify those which are also involved in binding interactions according to the homology model.

**Table 16:** Estimate of the overall binding free energies of some well-known substrates and inhibitors of P-gp using the experimentally solved cryo–electron microscopy structure of *h*P-gp (PDB ID: 6QEX).

Name	CDOCKER Binding Energy (kcal/mol)	GOLD Binding Energy (kcal/mol)
CsA ^1^	−133.400	−149.461
AM ^2^	−67.974	−64.093
DOX ^3^	−133.886	−149.007
DIG ^4^	−89.074	−111.314
LPM ^5^	−88.284	−73.676
RMP ^6^	−104.424	−142.062
VER ^7^	−80.413	−70.402
CAR ^8^	−70.329	−68.845
VPA ^9^	−29.039	−30.858
BU ^10^	−43.311	−37.354
GEN ^11^	−101.718	−99.725
APD ^12^	−69.750	−89.721
PQ ^13^	−112.572	−100.914

^1^ Cyclosporine A; ^2^ amiodarone; ^3^ doxorubicin; ^4^ digoxin; ^5^ loperamide; ^6^ rifampin; ^7^ verapamil; ^8^ carvedilol; ^9^ valproic acid; ^10^ busulfan; ^11^ gentamicin; ^12^ pamidronate; ^13^ paraquat.

## Supplementary Materials

The following are available online at https://www.mdpi.com/1422-0067/21/11/4058/s1, Figure S1. Alignment of the *h*P-gp sequence with the different templates used: (a) alignment with the sequence of *m*P-gp (PDB ID: 4M1M); (b) alignment with the sequence of *m*P-gp (PDB IDs: 4M1M, 5KO2, 5KOY, 3G61, 3G5U) ); (c) alignment with the sequence of *m*P-gp (PDB IDs: 6FN4, 4M1M, 5KPI, 4M2S, 5KO2, 3G60) and the sequence of C. elegans P-gp (PDB ID: 4F4C), Figure S2. (a) Distribution of the obtained ligand poses (yellow) in the experimentally solved cryoEM structure of *h*P-gp (PDB ID: 6QEX); (b) View from the extracellular side of the protein looking into the internal chamber, Figure S3. 3D view of the interactions of the top-ranked poses obtained with the CDOCKER algorithm in the experimentally solved cryoEM structure of *h*P-gp (PDB ID: 6QEX), Figure S4. Top-ranked binding poses obtained by re-docking calculations of the co-crystallized ligand PDBE-100 into its defined binding pocket in the homology model of *h*P-gp using: (a) CDOCKER algorithm; (b) GOLD algorithm, Figure S5. Top-ranked binding poses obtained by re-docking calculations of the cryoEM ligand (Taxol) into its defined binding pocket in the cryoEM structure *h*P-gp using: (a) CDOCKER algorithm; (b) GOLD algorithm, Figure S6. Ramachandran plot of the truncated *h*P-gp selected model taking into account only the TMDs region, Table S1. Ligand– P-gp interaction types and amino acid residues involved in the binding of some well-known substrates and inhibitors of P-gp, Table S2. Root-mean-square deviation (RMSD) values in Å calculated by spatial comparison (heavy atoms) between the experimentally determined co-crystallized ligand’s conformation (PBDE-100) and its top-ranked dock poses, generated by the performed re-docking calculations using the homology model, Table S3. Root-mean-square deviation (RMSD) values in Å calculated by spatial comparison (heavy atoms) between the experimentally determined cryoEM ligand’s conformation (Taxol) and its top-ranked dock poses, generated by the performed re-docking calculations using the cryoEM structure of *h*P-gp, Table S4. Comparison of the Verify 3D, ERRAT and PROVE Scores of the selected model to perform the molecular docking calculations.
