# Supplementary material for: Homology Modeling of the Human P-glycoprotein (ABCB1) and Insights into Ligand Binding through Molecular Docking Studies
Source: Int J Mol Sci. 2020 Jun 5;21(11):4058. doi: 10.3390/ijms21114058 (PMC7312539; doi:10.3390/ijms21114058)
Supplement: Supplementary file 1 [file ijms-21-04058-s001.pdf]

Supplementary Materials for:

Homology Modelling of the Human P-glycoprotein (ABCB1) and Insights into Ligand Binding Through Molecular Docking Studies

Liadys Mora Lagares [1,2,*], Nikola Minovski [1], Ana Yisel Caballero Alfonso [2,3], Emilio Benfenati[3], Sara Wellens[4], Maxime Culot[4], Fabien Gosselet[4] and Marjana Novič [1,*]

1 Theory Department, Laboratory for Cheminformatics, National Institute of Chemistry, 1000 Ljubljana, Slovenia; liadys.moralagares@ki.si, nikola.minovski@ki.si, marjana.novic@ki.si
2 Jožef Stefan International Postgraduate School, 1000 Ljubljana, Slovenia
3 Laboratory of Environmental Chemistry and Toxicology, Department of Environmental Health Sciences, Istituto di Ricerche Farmacologiche "Mario Negri" - IRCCS, 20156 Milano, Italy; ana.caballero@marionegri.it, emilio.benfenati@marionegri.it
4 Laboratoire de la Barrière Hémato-Encéphalique (LBHE), Univ. Artois, UR 2465, F-62300 Lens, France; sara_wellens@ens.univ-artois.fr, maxime.culot@univ-artois.fr, fabien.gosselet@univ-artois.fr
* Correspondence: marjana.novic@ki.si, liadys.moralagares@ki.si; Tel.: +386-01-476-0253

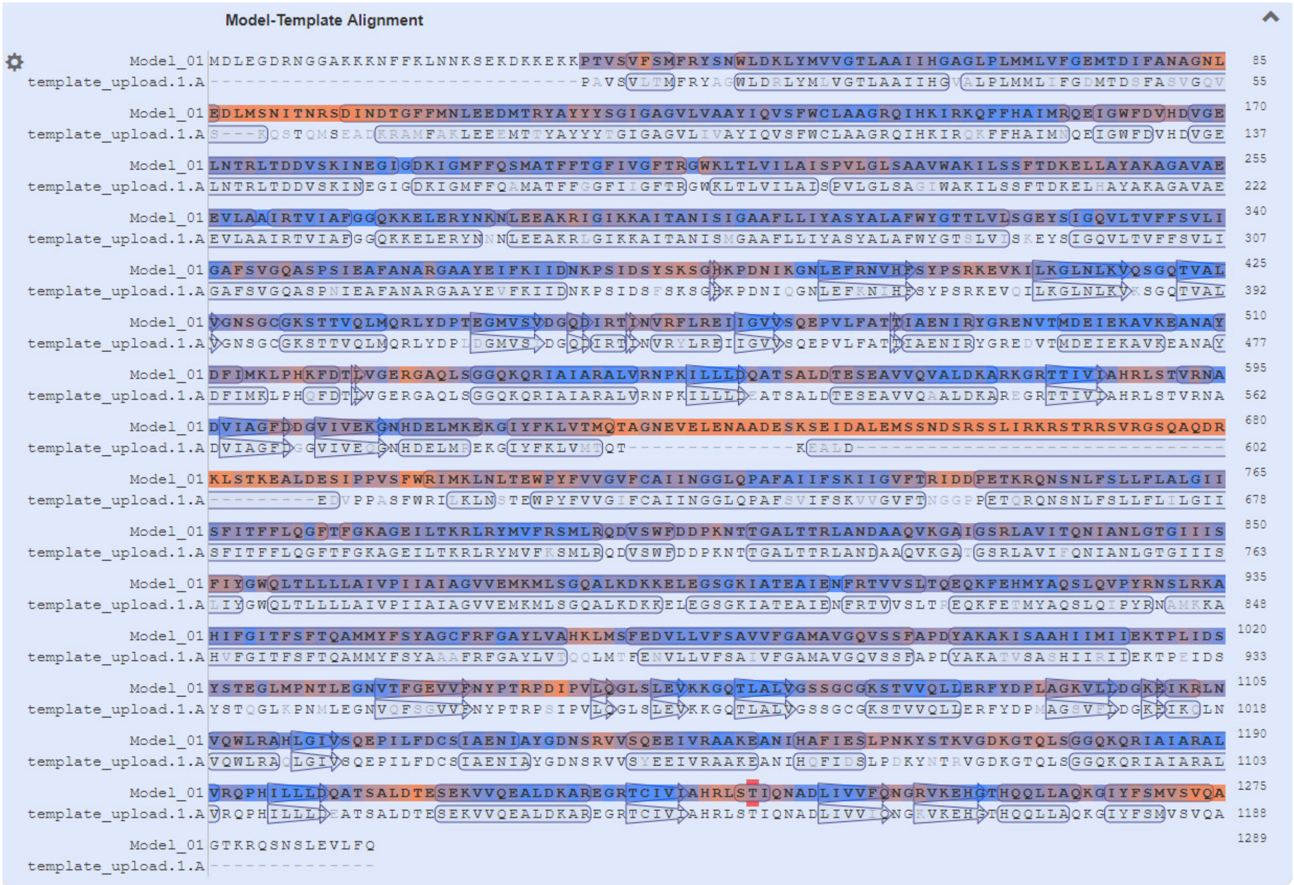

(**a**)

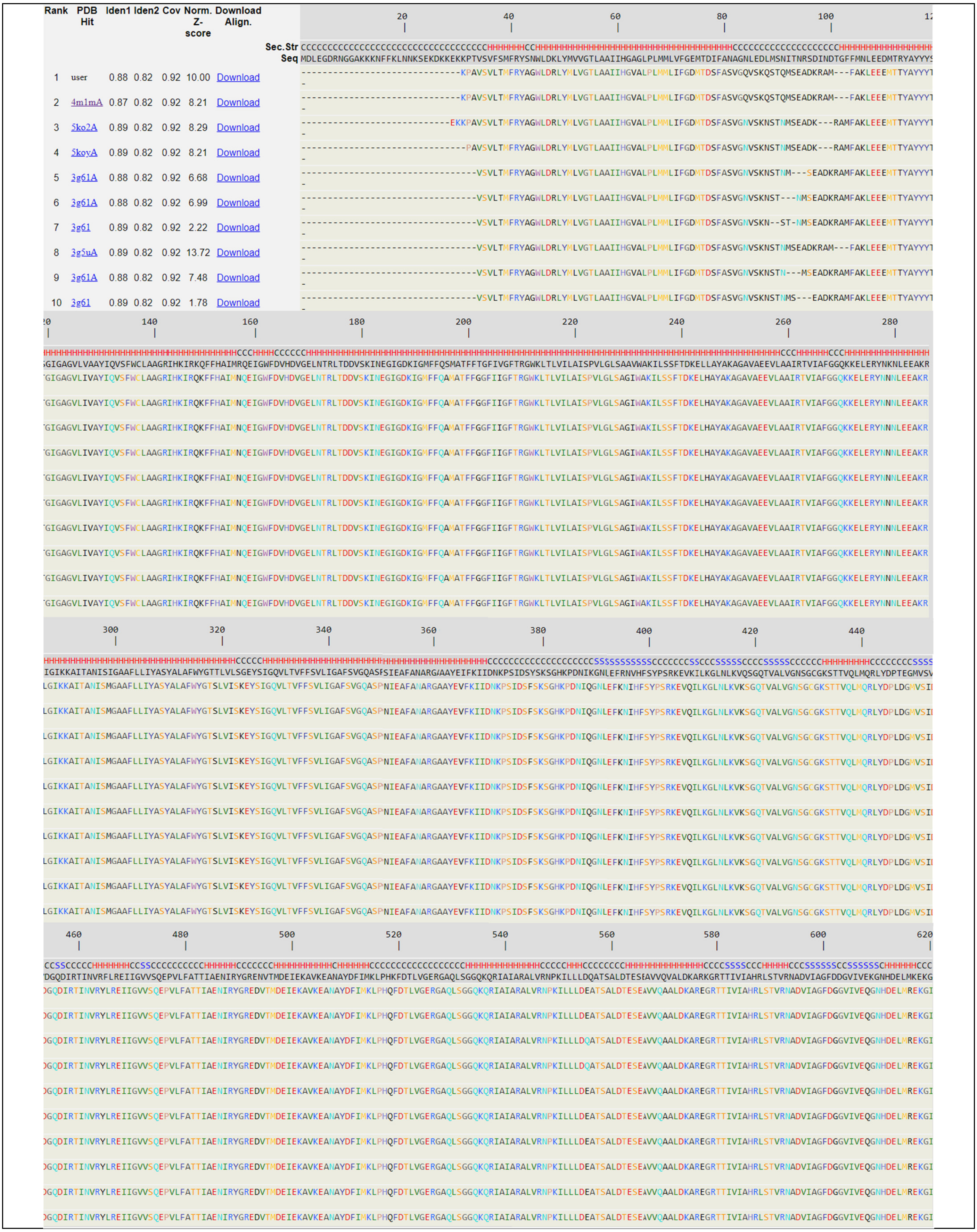
Rank
PDB Hit
Iden1
Iden2
Cov
Norm. Z-score
Download Align.
Sec.Str
Seq
1 user 0.88 0.82 0.92 10.00 Download
2 4m1mA 0.87 0.82 0.92 8.21 Download
3 5ko2A 0.89 0.82 0.92 8.29 Download
4 5koyA 0.89 0.82 0.92 8.21 Download
5 3g61A 0.88 0.82 0.92 6.68 Download
6 3g61A 0.88 0.82 0.92 6.99 Download
7 3g61 0.89 0.82 0.92 2.22 Download
8 3g5uA 0.89 0.82 0.92 13.72 Download
9 3g61A 0.88 0.82 0.92 7.48 Download
10 3g61 0.89 0.82 0.92 1.78 Download

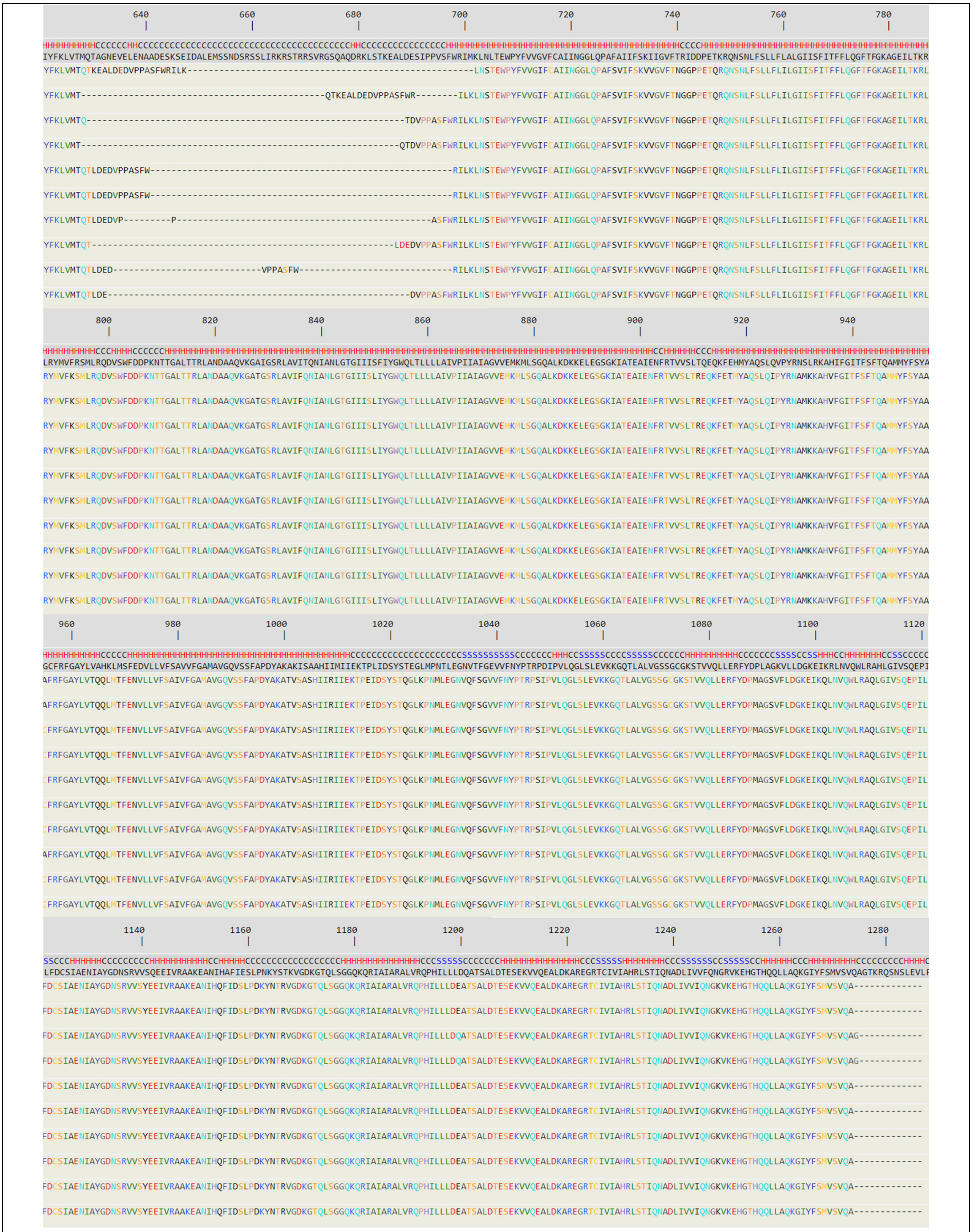
640 660 680 700 720 740 760 780
800 820 840 860 880 900 920 940
960 980 1000 1020 1040 1060 1080 1100 1120
1140 1160 1180 1200 1220 1240 1260 1280

(b)

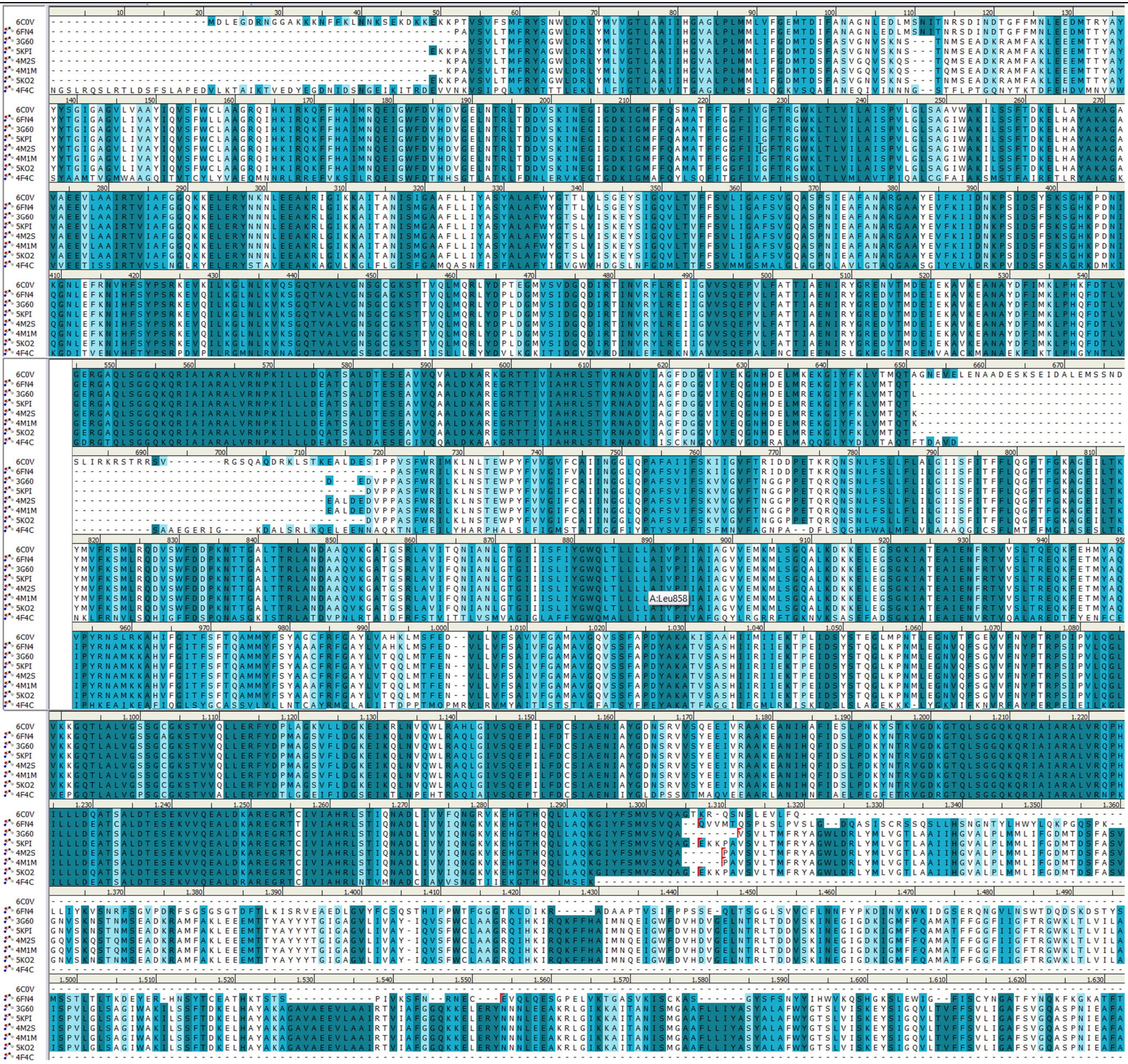

(**c**)

Figure S1. Alignment of the *h*P-gp sequence with the different templates used: (**a**) alignment with the sequence of *m*P-gp (PDB ID: 4M1M); (**b**) alignment with the sequence of *m*P-gp (PDB IDs: 4M1M, 5KO2, 5KOY, 3G61, 3G5U)); (**c**) alignment with the sequence of *m*P-gp (PDB IDs: 6FN4, 4M1M, 5KPI, 4M2S, 5KO2, 3G60) and the sequence of *C. elegans* P-gp (PDB ID: 4F4C).

Table 1. Ligand–P-gp interaction types and amino acid residues involved in the binding of some well-known substrates and inhibitors of P-gp. The numbers in parenthesis indicate the number of interactions in which the residue is involved.

Name	Hydrogen Bond	Alkyl	π-Sigma	π-Alkyl	π-π	π-Sulphur	π-Lone Pair
CsA[1]	N721, Q838, F303, F994, A987, Q990	A987 (2), V991 (3), L339 (3), I306	-	F303, Y310, F343 (3)	-	-	-
AM[2]	-	A311, I340, M986	F759	Y307, Y310, F336, F759	F728, Y310	-	Y310
DOX[3]	A980, S979	A729	-	-	-	-	F983
DIG[4]	Q725	-	F983	Y310, F314, F336, F 343, F728 (2), F732, F883 (2)	-	-	-
LPM[5]	Y310	-	F728	F728, F983, L339, I340	F314, F732, F759	-	F983
RMP[6]	F728, S979, I306	-	-	Y307 (2), F336 (2), F343, F728 (2), F732(2), F983 (3), M986	-	-	-
VER[7]	A987 (2)	I306, L339	-	F343, A987	Y953	M68	-
CAR[8]	F983	-	F732	I735	F314, F336, F732, F759, F978	-	-
VPA[9]	F728	-	-	Y307, F314, F732, F759	-	-	-
BU[10]	Y307, F732, F759	-	-	-	-	F732, Y310	-
GEN[11]	-	I340	-	-	-	-	-
APD[12]	N721, Q725	-	-	-	-	-	F303
PQ[13]	S979	-	-	-	F728 (2), F732		

[1] Cyclosporine A. [2] Amiodarone. [3] Doxorubicin. [4] Digoxin. [5] Loperamide. [6] Rifampin. [7] Verapamil. [8] Carvedilol. [9] Valproic Acid. [10] Busulfan. [11] Gentamicin. [12] Pamidronate. [13] Paraquat.

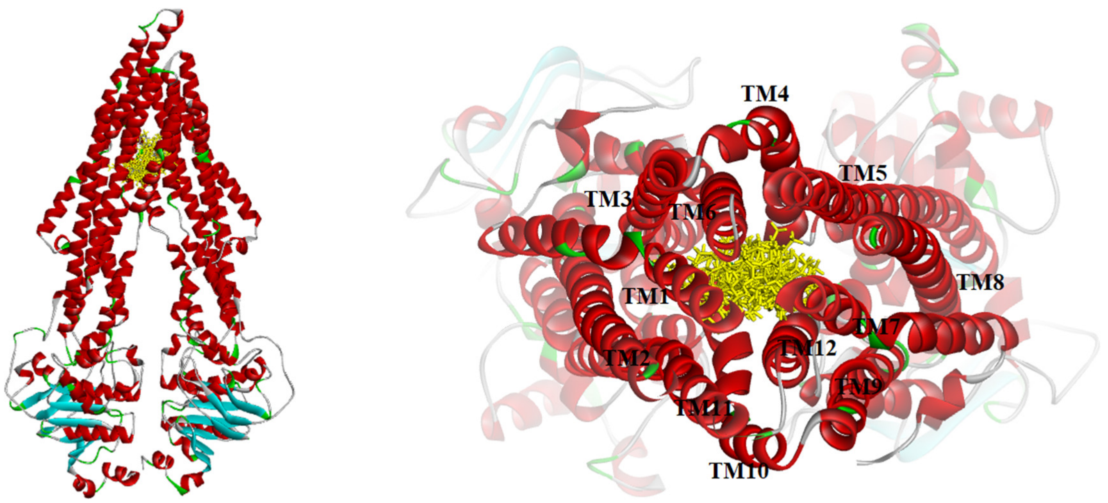

(**a**) (**b**)

Figure 2. (**a**) Distribution of the obtained ligand poses (yellow) in the experimentally solved cryoEM structure of *h*P-gp (PDB ID: 6QEX); (**b**) View from the extracellular side of the protein looking into the internal chamber. The colours representation is according to the secondary structure: helices are red, beta sheets are cyan, turns are green, and coils are white.

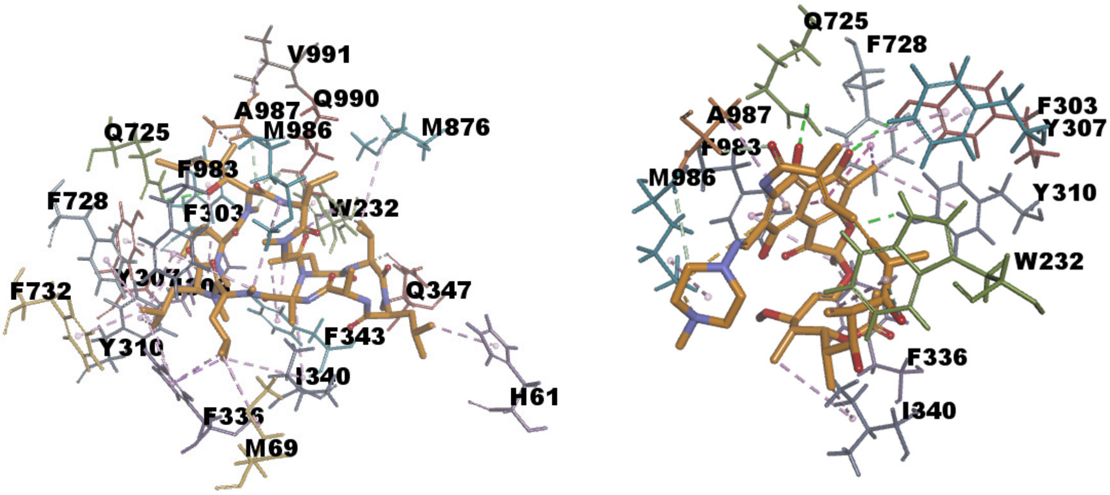

(**a**) (**b**)

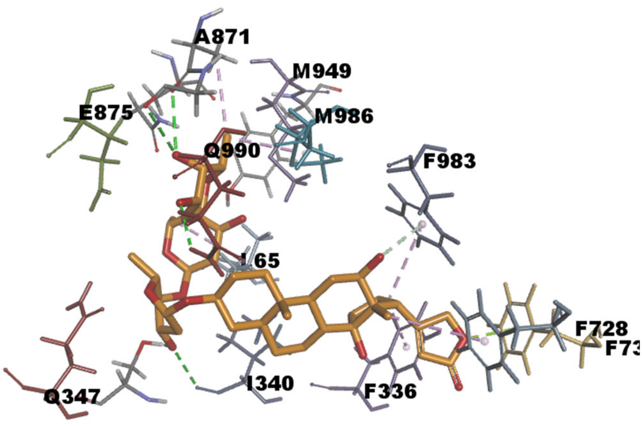
A871
M949
E875
M986
Q990
F983
F728
Q347
I340
F336

(**c**)

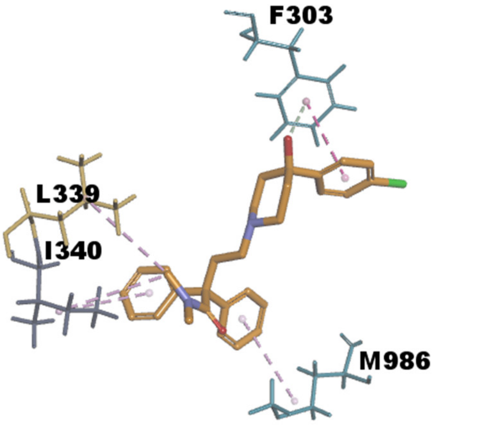
F303
L339
I340
M986

(**d**)

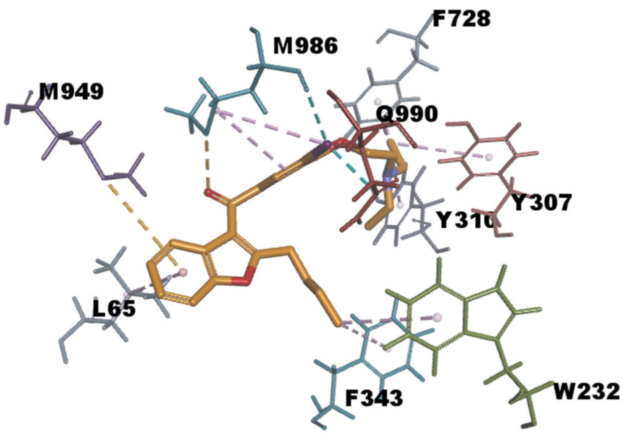
F728
M986
M949
Q990
Y307
Y310
L65
F343
W232

(e)

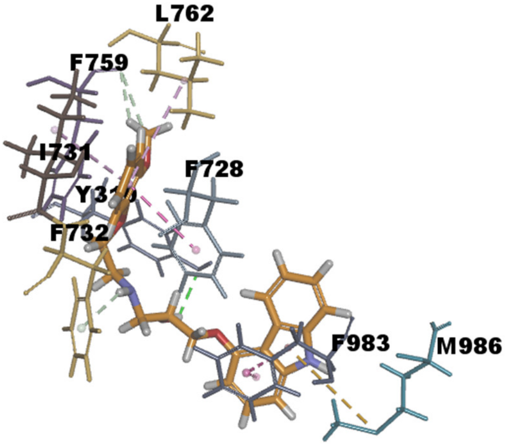
L762
F759
I731
F728
F732
F983
M986

(f)

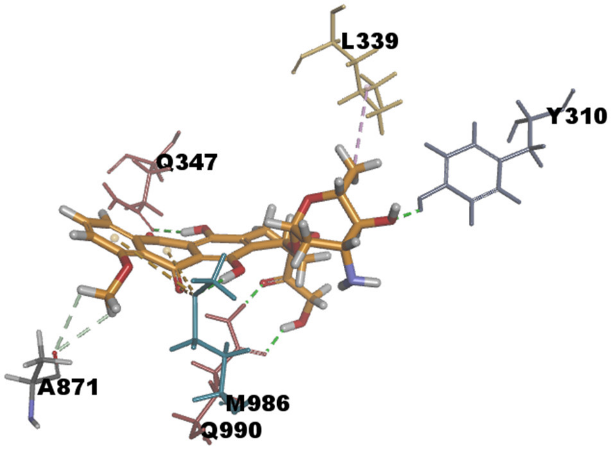
L339
Y310
Q347
A871
M986
Q990

(g)

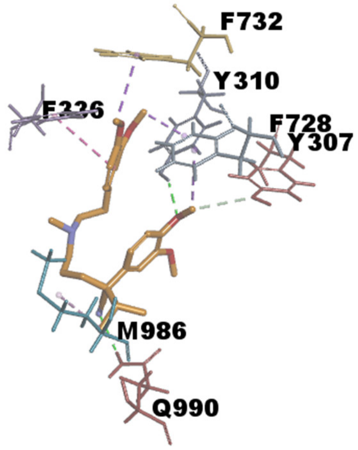
F732
Y310
F728
Y307
M986
Q990

(h)

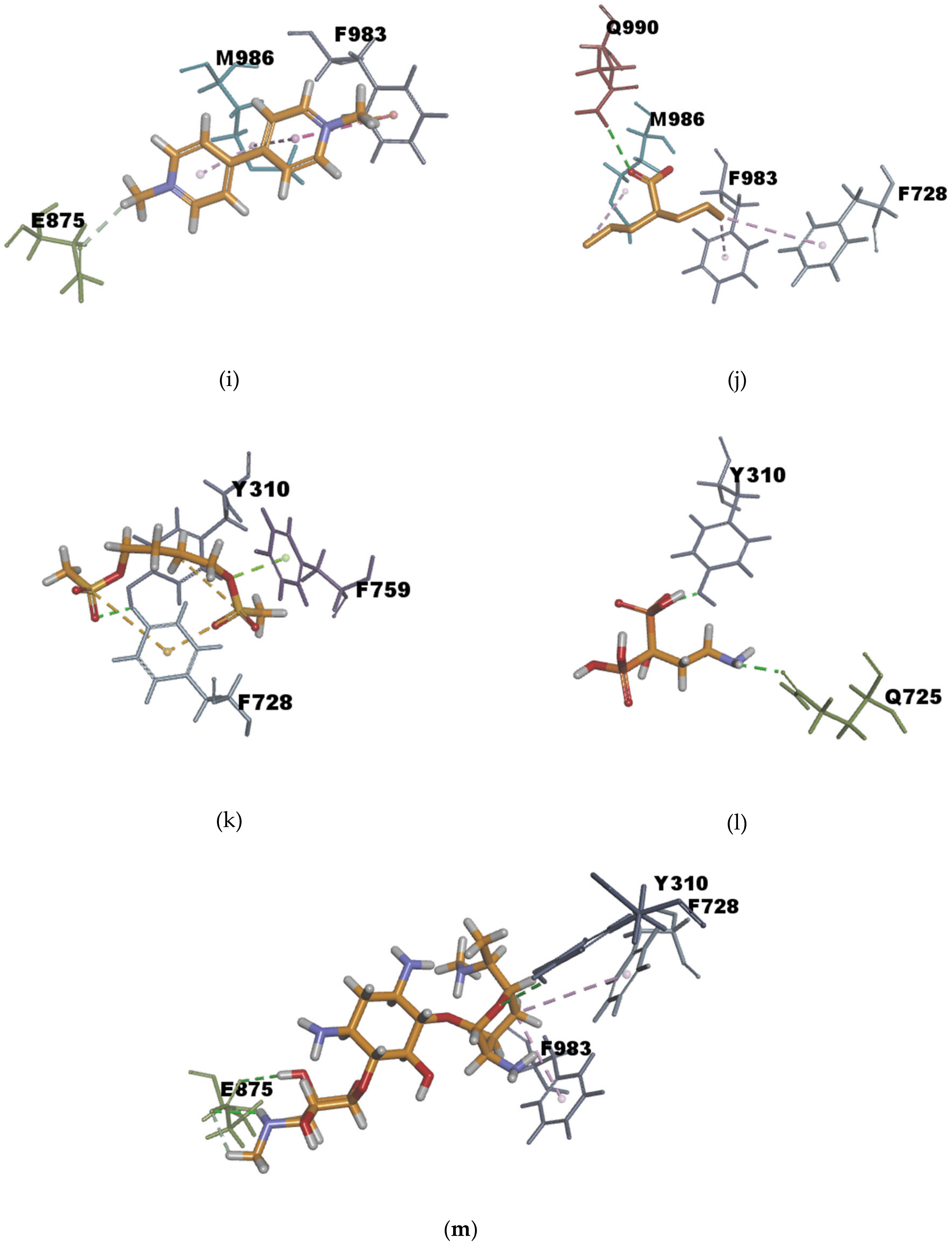

Figure 3. 3D view of the interactions of the top-ranked poses obtained with the CDOCKER algorithm in the experimentally solved cryoEM structure of *h*P-gp (PDB ID: 6QEX). (**a**) Cyclosporine A; (**b**) Rifampin; (**c**) Digoxin; (**d**) Loperamide; (**e**) Amiodarone; (**f**) Carvedilol; (**g**) Doxorubicin; (**h**) Verapamil; (**i**) Paraquat; (**j**) Valproic Acid; (**k**) Busulfan; (**l**) Pamidronate; (**m**) Gentamicin. The green dotted lines represent a conventional hydrogen bond interaction, light green dotted lines represent a carbon hydrogen bond interaction, light rose dotted lines represent hydrophobic interactions, orange dotted lines represent π-sulphur interactions and sulphur-X interactions, cyan dotted line represents halogen interactions and fluorescent green represents π-lone pair interactions.

Table 2. Root-mean-square deviation (RMSD) values in Å calculated by spatial comparison (heavy atoms) between the experimentally determined co-crystallized ligand's conformation (PBDE-100) and its top-ranked dock poses, generated by the performed re-docking calculations using the homology model.

**Model**	**Homology Model**					
Ligand	PBDE-100					
Method	CDOCKER			GOLD		
Docked pose	Dock 1	Dock 2	Dock 3	Dock 1	Dock 2	Dock 3
RMSD (Å)	1.5697	1.6021	1.6427	0.5527	0.7988	0.8498

Table 3. Root-mean-square deviation (RMSD) values in Å calculated by spatial comparison (heavy atoms) between the experimentally determined cryoEM ligand's conformation (Taxol) and its top-ranked dock poses, generated by the performed re-docking calculations using the cryoEM structure of *h*P-gp.

**Model**	**cryoEM structure of *h*P-gp**					
Ligand	Taxol					
Method	CDOCKER			GOLD		
Docked pose	Dock 1	Dock 2	Dock 3	Dock 1	Dock 2	Dock 3
RMSD (Å)	1.2723	1.3208	1.4630	1.0283	1.1974	1.2669

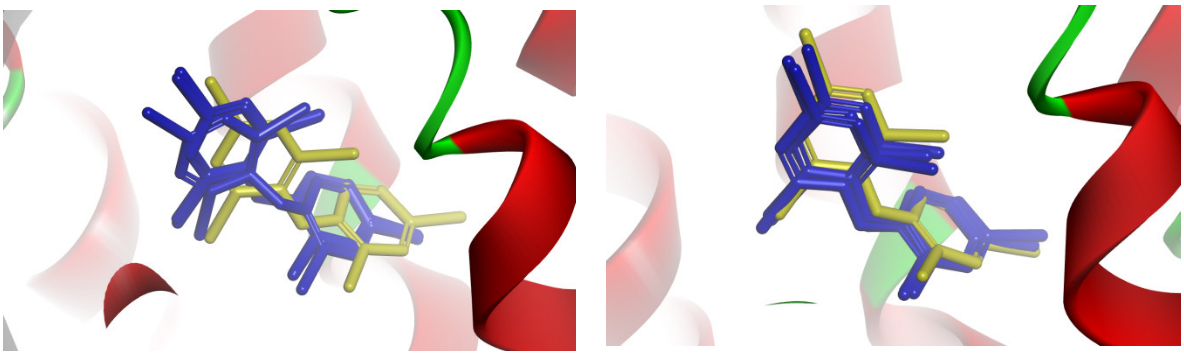

(**a**) (**b**)

Figure S4. Top-ranked binding poses obtained by re-docking calculations of the co-crystallized ligand PDBE-100 into its defined binding pocket in the homology model of *h*P-gp using: (**a**) CDOCKER algorithm; (**b**) GOLD algorithm. The experimental co-crystallized ligand is presented in solid yellow while the calculated top-ranked ligand poses are represented in solid blue (Table S2).

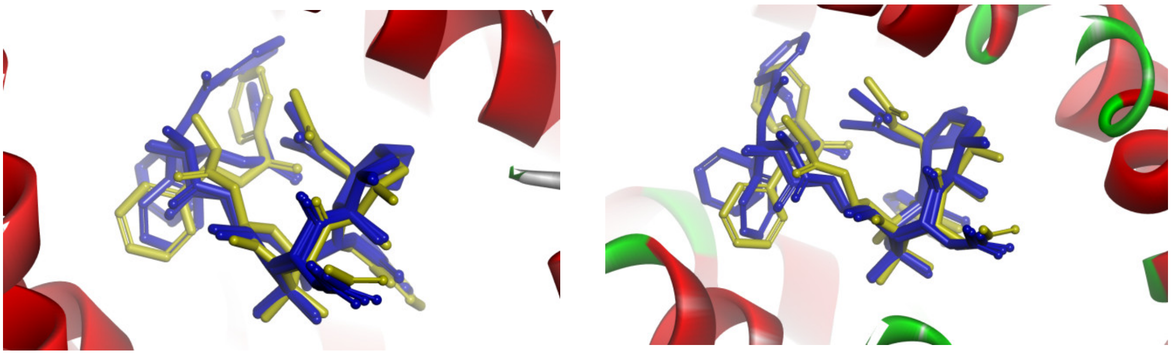

(**a**) (**b**)

Figure S5. Top-ranked binding poses obtained by re-docking calculations of the cryoEM ligand (Taxol) into its defined binding pocket in the cryoEM structure *h*P-gp using: (**a**) CDOCKER algorithm; (**b**) GOLD algorithm. The experimental cryoEM ligand is presented in solid yellow while the calculated top-ranked ligand poses are represented in solid blue (Table S3).

Table 4. Comparison of the Verify 3D, ERRAT and PROVE Scores of the selected model to perform the molecular docking calculations.

	**Model TMD**[1]	**Model IT**[2]	**PDB ID: 4M1M**[3]
Verify 3D	38.19%	63.41%	65.20%
ERRAT	95.1368	96.0884	86.5620
PROVE	5.0%	5.6%	0.0%

[1] Truncated *h*P-gp model (TMDs only). [2] Full-length I-TASSER *h*P-gp model. [3] Reference crystallographic structure of *m*P-gp

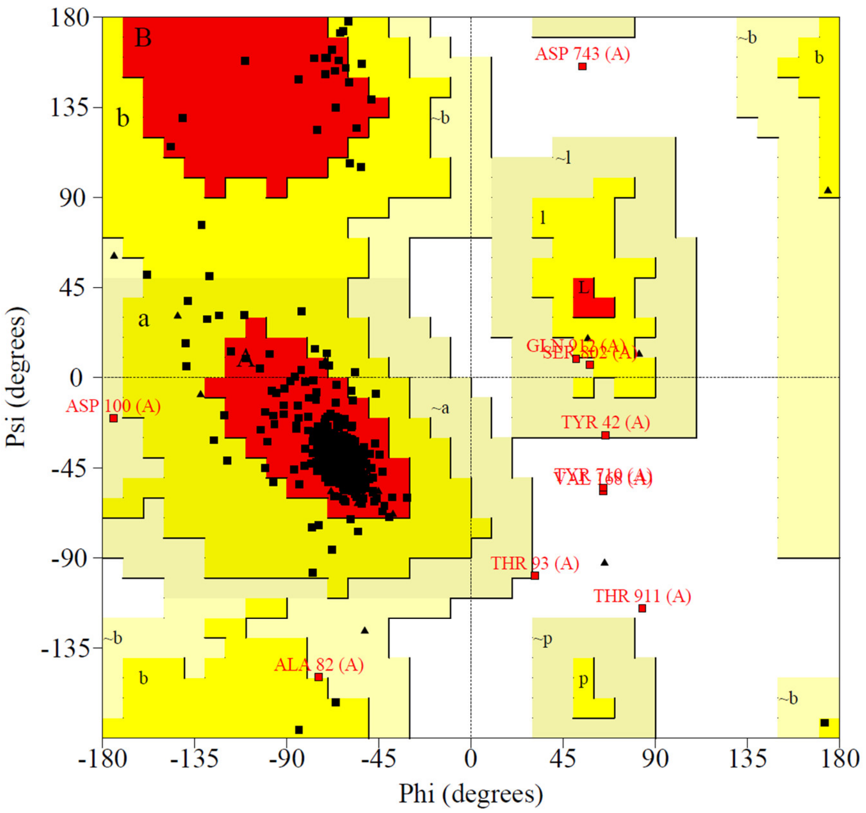

Plot statistics

Residues in most favoured regions [A,B,L]	542	92.6%
Residues in additional allowed regions [a,b,l,p]	33	5.6%
Residues in generously allowed regions [~a,~b,~l,~p]	5	0.9%
Residues in disallowed regions	5	0.9%

Figure S6. Ramachandran plot of the truncated *h*P-gp selected model taking into account only the TMDs region. The red, yellow and white areas represent the favoured, allowed and disallowed regions respectively.
